# Rapid Decline of Ceftazidime Resistance in Antibiotic-Free and Sublethal Environments Is Contingent on Genetic Background

**DOI:** 10.1093/molbev/msac049

**Published:** 2022-03-15

**Authors:** Sara Hernando-Amado, Pablo Laborda, José Ramón Valverde, José Luis Martínez

**Affiliations:** Centro Nacional de Biotecnología, CSIC, Madrid, Spain

**Keywords:** *Pseudomonas aeruginosa*, decline of antibiotic resistance, collateral sensitivity, fitness cost, compensatory evolution

## Abstract

Trade-offs of antibiotic resistance evolution, such as fitness cost and collateral sensitivity (CS), could be exploited to drive evolution toward antibiotic susceptibility. Decline of resistance may occur when resistance to other drug leads to CS to the first one and when compensatory mutations, or genetic reversion of the original ones, reduce fitness cost. Here we describe the impact of antibiotic-free and sublethal environments on declining ceftazidime resistance in different *Pseudomonas aeruginosa* resistant mutants. We determined that decline of ceftazidime resistance occurs within 450 generations, which is caused by newly acquired mutations and not by reversion of the original ones, and that the original CS of these mutants is preserved. In addition, we observed that the frequency and degree of this decline is contingent on genetic background. Our results are relevant to implement evolution-based therapeutic approaches, as well as to redefine global policies of antibiotic use, such as drug cycling.

## Introduction

The effectiveness of antibiotics is currently compromised by the emergence and spread of antibiotic resistance (AR). This evolutionary process presents two sequential steps: emergence and fixation. Although emergence is the consequence of selection, fixation of AR mainly depends on the fitness costs associated with resistance and on the chances of compensating these costs by acquiring secondary mutations.

It has been generally accepted that mutational acquisition of AR may impose a functional/metabolic burden, enhancing the probability of the resistant mutants to be outcompeted by a wild-type susceptible strain in antibiotic-free environments (Andersson and Levin 1999; Bjorkman and Andersson 2000; Martinez and Baquero 2000; Andersson 2006; Baquero et al. 2009; Balasubramanian et al. 2012; Tian et al. 2016). However, some AR mutations are cost-free (Wasels et al. 2015) or may even produce an increased fitness in antibiotic-free environments (Skurnik et al. 2013; Durão et al. 2015; Maharjan and Ferenci 2017). Besides, mutations that compensate fitness costs are frequently selected more likely than genetic reversion by restoration of the wild-type allele (Levin et al. 2000). This means that simply restricting the use of antibiotics would be insufficient to solve the AR problem (Schrag et al. 1997; Enne et al. 2001; Andersson and Hughes 2010; Brolund et al. 2010).

Despite the above, a rapid decline of AR has been observed using adaptive laboratory evolution (ALE) assays on antibiotic-free medium (Dunai et al. 2019). Nevertheless, resistance loss associated with compensatory evolution was observed to be drug specific. In addition, it is possible that AR decline may depend on both, the initial fitness cost and the original genetic background in which those fitness costs are compensated, because epistatic interactions, in addition to restricting AR evolution (Trindade et al. 2009; Ward et al. 2009; Salverda et al. 2011; Kryazhimskiy et al. 2014; Vogwill et al. 2016; Knopp and Andersson 2018; Hernando-Amado et al. 2019), may also affect compensatory evolution, something that has not been studied so far. Further, it is also possible that AR decline may depend on the environment, as fixation of AR does. It is known that high-cost AR genetic variations are preserved or rapidly outcompeted depending on whether or not antibiotics are present in the environment. Because of that, most studies in the field have focused on the study of AR evolution on antibiotic-free environments or inhibitory (hereafter dubbed lethal, to simplify) concentrations of antibiotics. However, subinhibitory (hereafter dubbed sublethal, to simplify) concentrations can also select antibiotic resistant mutants (Sanz-García et al. 2021) and they can be present in certain clinical situations (e.g., incomplete treatments or limited drug accessibility) (Baquero and Negri 1997). Therefore, it is possible that sublethal concentrations of antibiotics may be relevant during the evolutionary compensation of fitness costs, a feature that has not been addressed yet.

In a previous work, we determined to what extent the presence of pre-existing AR mutations may affect the robustness of ceftazidime resistance evolution in *Pseudomonas aeruginosa*. For this purpose, we submitted different *P. aeruginosa* resistant mutants, presenting either single mutations, NfxB or ParR, or multiple resistance mutations, MDR6, to short-term evolution in the presence of ceftazidime (Hernando-Amado et al. 2020). The evolved populations presented large chromosomal deletions leading to increased ceftazidime minimal inhibitory concentration (MIC) and reduced tobramycin MIC (Hernando-Amado et al. 2020), antibiotics that form part of usual therapies against *P. aeruginosa* (Cheer et al. 2003). Therefore, we proposed that this robust phenotypic convergence toward collateral sensitivity (CS) (Szybalski and Bryson 1952; Pal et al. 2015), by which acquisition of resistance to a drug (i.e., ceftazidime) increases susceptibility to another (i.e., tobramycin), could be exploited to rationally counteract *P. aeruginosa* infections, alternating ceftazidime with tobramycin. However, the stability of ceftazidime resistance and CS to tobramycin once ceftazidime is removed, where compensatory evolution of fitness costs may occur, was not determined. Furthermore, we did not study the evolution of tobramycin resistance in the presence of tobramycin sublethal concentrations and if it could lead to CS to ceftazidime, a possibility supported by previous findings showing the existence of reciprocal CS between aminoglycosides and β-lactams (Barbosa et al. 2019).

**Fig. 1.**
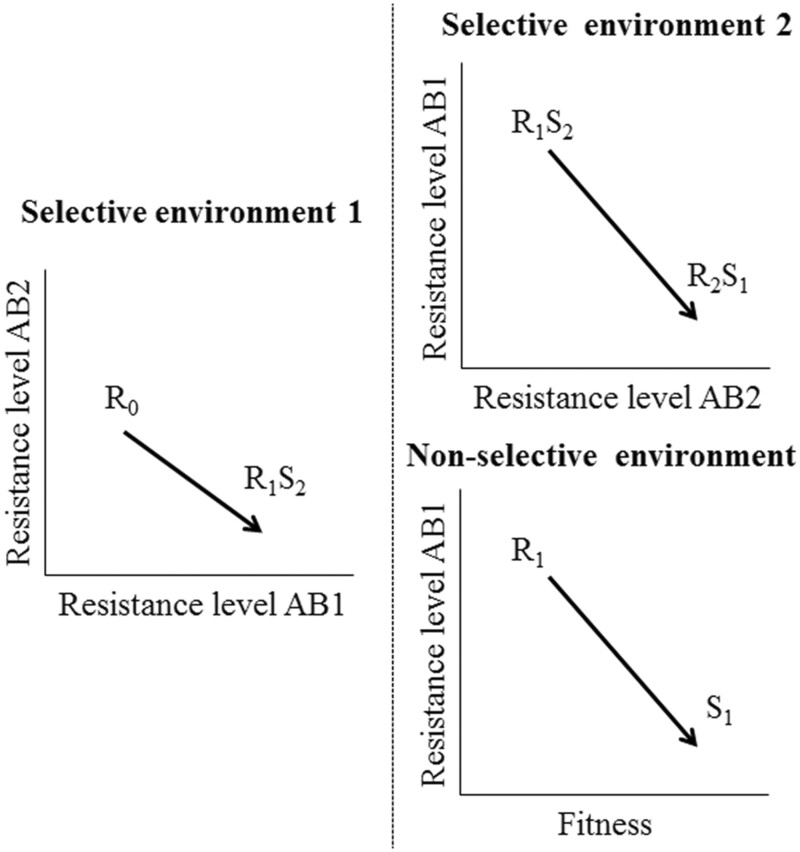
Conceptual figure representing phenotypic reversion of drug resistance. Departing from an initial AR phenotype (R_0_), exposure to a first antibiotic (AB1) may select resistance to that first drug (R_1_) and CS (S_2_) to a second antibiotic (AB2) (selective environment 1). After that first step, which leads to an organism resistant to the first drug and susceptible to the second antibiotic (R_1_S_2_), reversion of resistance acquired may occur in sublethal concentrations of AB2 that select resistance to this antibiotic and CS to AB1 (R_2_S_1_) (selective environment 2). This decline of resistance may also occur in antibiotic-free environments or sublethal antibiotic concentrations that do not select resistance, when fitness costs of resistance to the first drug are compensated (nonselective environment), hence moving from low fitness and resistance to AB1 (R_1_) to a high fitness related susceptible phenotype (S_1_).

In this work, we analyze the possible decline of ceftazidime resistance during 8 weeks of ALE experiments in two different environments: 1) antibiotic-free medium (e.g., drug restriction periods) and 2) sublethal tobramycin concentrations (e.g., concentrations resulting from tobramycin and ceftazidime alternation in sequential therapies). Our hypothesis was that fitness cost of ceftazidime resistant mutants could drive compensatory evolution in nonselective environments (antibiotic-free or tobramycin sublethal concentrations that do not select resistance) and a decline of ceftazidime resistance (see “nonselective environment” in conceptual fig. 1). In particular, we raised the question of the extent to which both, genetic background and initial fitness costs could shape compensatory evolution and the decline of resistance, if it occurred. Besides, we analyzed if sublethal tobramycin concentrations able to select resistance in each genetic background could also lead to CS to ceftazidime (see “selective environment 2” in conceptual fig. 1). Finally, we studied the stability of CS to tobramycin of the analyzed ceftazidime resistant mutants in antibiotic-free environments, because compensatory evolution may not only cause a decline of ceftazidime resistance but also variations in trade-offs associated with this phenotype, as it is CS to tobramycin.

## Results

### Characterization of NfxB-CAZ, ParR-CAZ, and MDR6-CAZ Ceftazidime Resistant Mutants

**Fig. 2.**
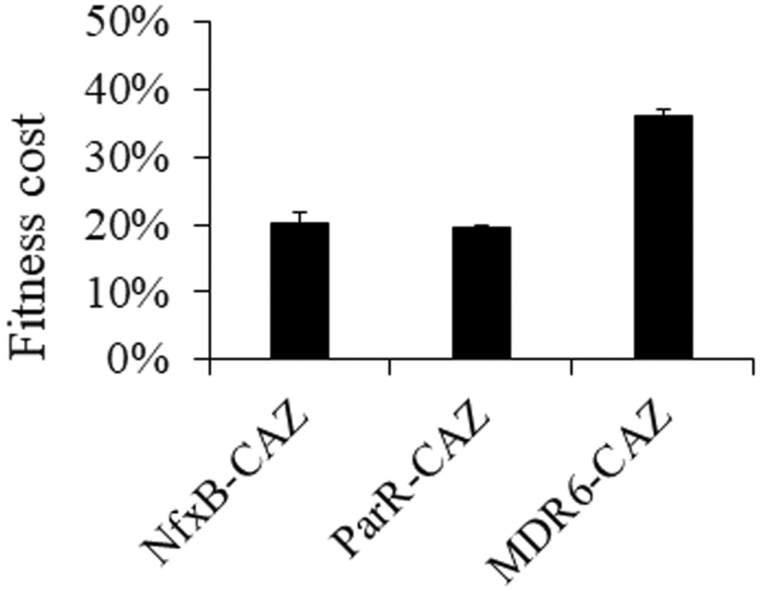
Ceftazidime-resistant mutants present a high fitness cost in antibiotic-free medium. Growth curves of the resistant mutants, before and after ceftazidime evolution, were recorded in antibiotic-free medium (LB). The fitness (*W*) of each strain was measured as the area under the growth curve. Fitness cost of each ceftazidime resistant mutants respect to its parental strain was calculated, using the equation: 1 − (*W*_mutant_/*W*_parental strain_) and was expressed as percentage. Acquisition of resistance to ceftazidime is associated with fitness cost in antibiotic-free medium. Error bars indicate standard deviations from three different replicates.

A clone from each of the three different ceftazidime resistant/tobramycin hyper-susceptible populations (NfxB, ParR, and MDR6) previously obtained (Hernando-Amado et al. 2020), was isolated (hereafter dubbed NfxB-CAZ, ParR-CAZ, or MDR6-CAZ, to simplify). The three clones were subjected to whole-genome sequencing. Consistent with previous information concerning the genotype of the populations containing these mutants, we identified three genetic events in NfxB-CAZ, including a large chromosomal deletion and two single-nucleotide polymorphisms (SNPs), three genetic events in ParR-CAZ, including a large chromosomal deletion and two SNPs, and seven genetic events in MDR6-CAZ, including a large chromosomal deletion, and six SNPs (supplementary table 1, Supplementary Material online). Given that the three ceftazidime resistant clones lacked large genomic regions, it was sensible to hypothesize that they could present high fitness costs when grown in the absence of antibiotics. In addition, it is important to notice that MDR6-CAZ and ParR-CAZ mutants presented a SNP in *rpoB*, that encodes the subunit β of the RNA polymerase (RNAP) (Villain-Guillot et al. 2007), and in *ampR*, which encodes a LysR-type transcriptional regulator (LTTR) that regulates the expression of the β-lactamase AmpC (Kong et al. 2005; Balcewich et al. 2010), respectively (supplementary table 1, Supplementary Material online), respectively. Mutations in the genes encoding these two proteins are known to reduce bacterial fitness (Severinov et al. 1993; Qi et al. 2014; Perez-Gallego et al. 2016; Balbontin et al. 2021). We, therefore, compared the fitness of NfxB-CAZ, ParR-CAZ, or MDR6-CAZ mutants, before and after short-term evolution on ceftazidime, in antibiotic-free medium (fig. 2). For that, we estimated fitness of each mutant and parental strain as the area under the growth curve recorded in antibiotic-free medium and we calculated fitness costs of each ceftazidime resistant mutants respect to its parental strain (see Materials and Methods). The three ceftazidime resistant mutants presented a reduction of fitness with respect to their parental strains, from 20% in the case of NfxB-CAZ or ParR-CAZ, up to 40% in the case of MDR6-CAZ. Given the strength of the fitness costs, it was worth thinking that these ceftazidime resistant mutants might acquire compensatory mutations, which would reduce the burden associated with ceftazidime resistance, in antibiotic-free, nonselective environments. In addition, it might be possible that some of the newly acquired compensatory mutations could produce a reversion or decline of resistance. Since these ceftazidime resistant mutants presented CS to tobramycin (supplementary table 2, Supplementary Material online), being possible to alternate ceftazidime with tobramycin (Hernando-Amado et al. 2020), it was also of interest to know the range of tobramycin sublethal concentrations that could select for tobramycin resistance, eventually altering the pattern of compensatory evolution.

### Evolution of Ceftazidime and Tobramycin Resistance on Antibiotic-Free and Sublethal Tobramycin Environments

To analyze changes in ceftazidime and tobramycin susceptibility upon evolution in antibiotic-free and sublethal tobramycin environments, we performed an 8-week ALE assay with the ceftazidime resistant mutants NfxB-CAZ, ParR-CAZ, and MDR6-CAZ, using four replicates of each genetic background, in four different environments: antibiotic-free (12 populations), 1/4 of tobramycin MIC (12 populations), 1/8 of tobramycin MIC (12 populations), and 1/16 of tobramycin MIC (12 populations). This resulted in a total of 48 independently evolved populations. The populations were propagated for 56 days, approximately 450 generations, by diluting 1% of the saturated cultures into fresh medium every day. The tobramycin and ceftazidime MICs for each population were determined every 7 days during the 56 days of the experiment (supplementary tables 2 and 3, Supplementary Material online, respectively).

**Fig. 3.**
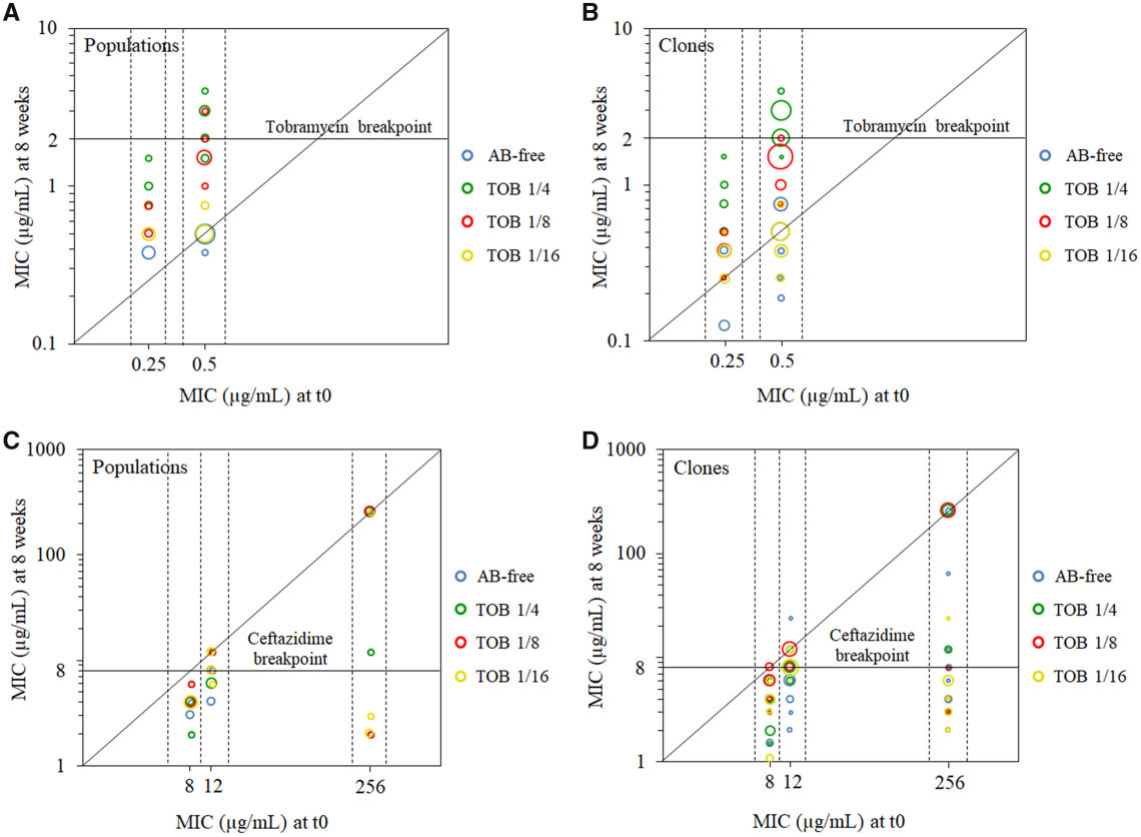
Impact of antibiotic-free and tobramycin sublethal environments on AR level. (*A* and *B*) The figure shows the MICs (µg/ml) to tobramycin (log10-scale) of each starting genetic background at time zero (*t*_0_) and the corresponding MIC (µg/ml) acquired in (*A*) the populations or (*B*) their isolated clones, after 8 weeks of ALE. Each of the points represents a population (in *A*) or clone (in *B*); and the colors indicate different treatments: AB free, 1/4 of tobramycin MIC, 1/8 of tobramycin MIC, and 1/16 of tobramycin MIC. The size of the points is proportional to the number of clones/populations within the same treatment presenting the same value. Raw MIC values appear in supplementary tables 2, 3, and 5, Supplementary Material online. Points above the diagonal line indicate cases with an increase of tobramycin resistance level after resistance evolution. Points above the horizontal line indicate cases with a tobramycin MIC above the clinical breakpoint (2 µg/ml). 1/4 of tobramycin MIC was able to select mutants presenting a MIC above the clinical breakpoint (4 out of 12 populations and 18 out of 48 clones), whereas 1/8 of MIC did in just 1 out of 12 population and none of the 48 clones (see supplementary tables 2 and 5, Supplementary Material online). (*C* and *D*) The figures show MIC (µg/ml) to ceftazidime (log10-scale) of each starting genetic background at time zero (*t*_0_) and the corresponding MIC (µg/ml) acquired in the populations (*C*) or their isolated clones (*D*) after 8 weeks of ALE. Each of the points represents a population (in *C*) or clone (in *D*); and the colors indicate different treatments: AB free, 1/4 of tobramycin MIC, 1/8 of tobramycin MIC, and 1/16 of tobramycin MIC. The size of the points is proportional to the number of clones/populations within the same treatment presenting the same value. Raw MIC values appear in supplementary tables 2, 3, and 5, Supplementary Material online. Points below the diagonal line indicate cases with a decrease of ceftazidime resistance level after compensatory or tobramycin resistance evolution. Points below the horizontal line indicate cases with a ceftazidime MIC below the clinical breakpoint (8 µg/ml). For mixed populations, MIC value of the susceptible subpopulation was represented (see supplementary table 3, Supplementary Material online). Most of the populations (35 out of 48) and clones (145 out of 192) showed a decline of resistance after 8 weeks of ALE on both, antibiotic-free or tobramycin sublethal environments. Importantly, only 25% of the clones presented a ceftazidime MIC above the clinical breakpoint. 75.5% of clones presented a reduction of ceftazidime MIC, being the degree of decline dependent on the genetic background: up to 128-fold in ParR-CAZ, 10.7-fold in NfxB-CAZ, and 6-fold in MDR6-CAZ.

Statistical analysis of the data, using Multi- and One-Way ANOVA with Welch correction followed by Tukey HSD multiple comparisons, revealed the existence of significant differences when considering each factor or combination thereof (*P* < 0.005 in all cases). Notably, a decline of ceftazidime resistance was observed, within the 56 days of ALE, in 35 out of 48 populations (72%) from both, nonlethal and tobramycin sublethal environments (supplementary table 3, Supplementary Material online and fig. 3*C*). Decline of ceftazidime MIC was observed in 9 out of 12 populations evolved in antibiotic-free environment, 9 out of 12 populations evolved in 1/4 of tobramycin MIC, 7 out of 12 populations evolved in 1/8 of tobramycin MIC, and 10 out of 12 populations evolved in 1/16 of tobramycin MIC (supplementary table 3, Supplementary Material online). However, the degree of resistance decline varied, depending on the genetic background, being up to 128-fold in ParR-CAZ, up to 4-fold in NfxB-CAZ, and up to 3-fold in MDR6-CAZ. Because ANOVA showed that differences depending on genetic background and time of evolution were significant (*P* < 0.001), we carried an endpoint analysis, as described in Materials and Methods. This revealed significant reductions of ceftazidime MIC in NfxB-CAZ populations in all the growth conditions (*P* < 0.01), in MDR6-CAZ populations in all conditions except in 1/8 of tobramycin MIC, and in ParR-CAZ populations in 1/16 of tobramycin MIC (*P* < 0.05).

Our initial hypothesis was that a decline of ceftazidime resistance in antibiotic-free conditions might be possible, due to compensatory evolution of fitness costs associated with ceftazidime resistance, and that the presence of concentrations of tobramycin unable to select tobramycin resistance mutations would not impede this AR decline (see conceptual fig. 1). In addition, our hypothesis was that the newly selected tobramycin resistance mutations, acquired in the presence of selective concentrations of tobramycin, could also present CS to ceftazidime (see conceptual fig. 1), because reciprocal CS between aminoglycosides and β-lactams has been previously described (Barbosa et al. 2019). In these two situations, ceftazidime resistance could decline, something clinically relevant, allowing a switch back to ceftazidime after drug restriction periods or alternation of ceftazidime with tobramycin.

End-point analysis (comparing MIC levels at *t* = 0 and *t* = 56 days) revealed significant differences (*P* < 0.05) in the three parental, antibiotic resistant, strains when treated with 1/4 and 1/8 of tobramycin MIC but not with the lower sublethal concentration (1/16 of tobramycin MIC) or without antibiotic. In the absence of drugs, compensatory evolution led to the decrease of ceftazidime resistance, but CS to tobramycin was preserved (see supplementary table 2, Supplementary Material online). For its part, a progressive increase in the level of resistance to the antibiotic of selection was observed for the two higher sublethal tobramycin concentrations (1/4 and 1/8 of tobramycin MIC) for the three analyzed genetic backgrounds, suggesting either accumulation of mutations along the evolution or the displacement of low-level resistant mutants by higher-level ones. In all the genetic backgrounds (and all replicate populations) analyzed, 1/4 and 1/8 of tobramycin MIC resulted in a tobramycin resistance increase at the end of the ALE experiments, although the resistance levels acquired were significantly lower (*P* < 0.001, using Tukey HSD) in the presence of 1/8 of tobramycin MIC than in the presence of 1/4 of tobramycin MIC (supplementary table 2, Supplementary Material online and fig. 3*A*). This is in agreement with previous data from our laboratory that described, in a PA14 wild-type strain, a threshold of the sublethal selective window higher than 1/10 of tobramycin MIC (Sanz-Garcia et al. 2020). In fact, only the higher sublethal tobramycin concentration (1/4 of tobramycin MIC) was able to select mutants presenting a tobramycin MIC above the EUCAST clinical breakpoint (2 µg/ml) (fig. 3*A*). However, it is important to highlight that, even in these cases, the MICs reached by the mutants were not high; well below those described when the PA14 wild-type strain was submitted to tobramycin sublethal selection (Sanz-Garcia et al. 2018b). In addition, a very subtle, not statistically significant, increase was observed in the case of antibiotic-free medium or the lower tobramycin concentration tested (1/16 of tobramycin MIC) (supplementary table 2, Supplementary Material online). No significant increase of resistance was observed in 1/16 of tobramycin MIC in any of the NfxB-CAZ replicate populations, nor in 2 out of 4 ParR-CAZ replicate populations. However, a detectable increase was observed in 2 out of 4 ParR-CAZ replicate populations and MDR6-CAZ replicate populations. ANOVA showed that the level of tobramycin resistance significantly depends on genetic background (*P* < 0.001) and time of evolution (*P* < 0.01) for all treatments. Significant differences (*P* < 0.01) could be found among genetic backgrounds and treatments in subsequent Tukey’s test, indicating that the probability to select resistance at a particular sublethal tobramycin concentration depends on the genetic background (fig. 3*A*). These results support the influence of the genetic background on determining the sublethal selective concentration that selects resistance to tobramycin, something that had not been previously described.

Altogether, these results support that the alternation of ceftazidime with tobramycin for treating *P. aeruginosa* infections, that we previously suggested (Hernando-Amado et al. 2020), could be followed, if required, by a switch back to ceftazidime. Further, these results also suggest the possibility of alternating ceftazidime with periods of drug restriction, allowing a rapid decline of resistance, preserving tobramycin CS, and possibly avoiding the selection of resistance to other antibiotics, a feature that we study below.

### Evolution of Cross-Resistance and Collateral Sensitivity on Antibiotic-Free and Sublethal Tobramycin Environments

The ceftazidime resistant mutants NfxB-CAZ, ParR-CAZ, and MDR6-CAZ obtained after short-term evolution on ceftazidime presented CS, not only to tobramycin, but also to fosfomycin and tetracycline, and cross-resistance to aztreonam and imipenem (Hernando-Amado et al. 2020). To ascertain if the populations obtained after ALE on both, antibiotic-free or tobramycin sublethal environments, besides presenting a decline of ceftazidime MIC, could also present variations in the level of resistance to other antibiotics belonging to different structural families (fosfomycin, tetracycline, ciprofloxacin, chloramphenicol, aztreonam, and imipenem), MICs for these antibiotics were determined for the 48 populations and analyzed using ANOVA followed by post-hoc tests (Tukey for all vs. all and Dunnett with Hochberg correction for many to one). MIC changes were evaluated using EUCAST values, proportion tests and log-transformed confidence intervals, as described in Materials and Methods. An important, statistically significant, decrease of resistance to aztreonam (*P* < 0.01) and imipenem (*P* < 0.001) was observed, whereas the phenotypes of CS were maintained and no further change of susceptibility to other antibiotics was detected (supplementary table 4, Supplementary Material online). The case of imipenem is particularly remarkable, because decline of MIC was observed in 41 out of 48 populations (85%) from both, nonlethal and sublethal environments (supplementary table 4, Supplementary Material online) with 31 populations having values below the EUCAST breakpoint (*P* < 0.001, test for equality of proportions). Namely, imipenem MIC was reduced in 10 out of 12 populations evolved in antibiotic-free environment, 10 out of 12 populations evolved in 1/4 of tobramycin MIC, 11 out of 12 populations evolved in 1/8 of tobramycin MIC, and 10 out of 12 populations evolved in 1/16 of tobramycin MIC (supplementary table 4, Supplementary Material online). Altogether, our data support that compensatory evolution allows not only the decrease of resistance to ceftazidime but also of cross-resistance to other β-lactams while preserving CS to tobramycin, a trade-off originally associated with ceftazidime resistance evolution.

### Decline of Ceftazidime Resistance in Environments without Antibiotics or Containing Sublethal Concentrations of Tobramycin

As mentioned above, a reduction of ceftazidime MIC was observed in 72% of the populations evolved in environments without antibiotics (nonselective environments) or containing sublethal tobramycin concentrations (selective environments) (supplementary table 3, Supplementary Material online; fig. 3*C*). However, occasionally, subpopulations still resistant to ceftazidime were observed within the susceptible ones (supplementary table 3, Supplementary Material online). Therefore, we selected for further analysis four random clones from each of the 48 populations, hereafter dubbed as ParR, NfxB, or MDR6, followed by the number of the population from which they proceed and a letter (i.e., ParR 1a, a clone isolated from population ParR-CAZ 1). Ceftazidime and tobramycin MICs were measured in the 192 isolated clones (supplementary table 5, Supplementary Material online and fig. 3*B* and *D*) and analyzed checking EUCAST values and using ANOVA followed by post-hoc tests. Interestingly, we observed a ceftazidime MIC decrease in 76% of the clones and only 25% of all the clones presented ceftazidime MICs above the EUCAST clinical breakpoint (8 µg/ml) (fig. 3*D* and supplementary table 5, Supplementary Material online), depending on genetic background and selective environment (*P* < 0.001, Multi-Way ANOVA). It is important to highlight that, although we observed in most of these clones a decrease of ceftazidime MIC, it was not possible to know the degree of decrease for others, because their ceftazidime MICs were above the detection limit of the E-test strips (supplementary table 5, Supplementary Material online).

**Fig. 4.**
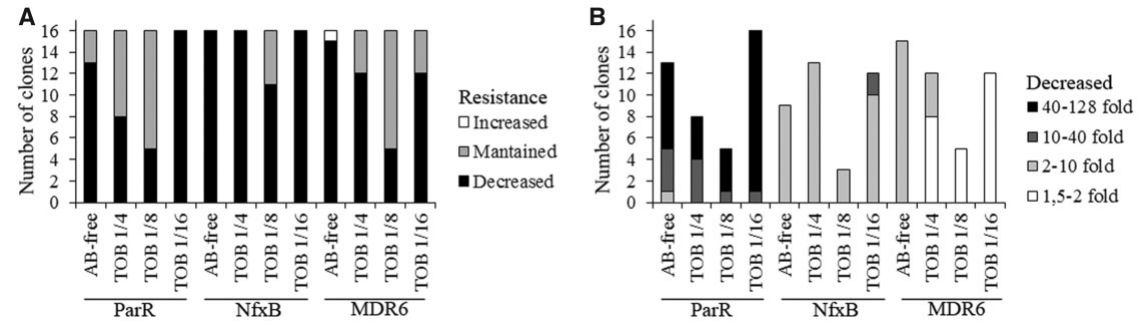
Decline of ceftazidime resistance in antibiotic-free and tobramycin sublethal environments depending on the genetic background. (*A*) The figure shows the number of clones that present decreased, maintained, or increased resistance to ceftazidime after ALE on antibiotic-free or tobramycin sublethal environments. Nonselective environments (antibiotic free and tobramycin 1/16) showed the higher number of ceftazidime declines (44 out of 48 clones, *P* < 0.05), followed by 1/4 of tobramycin selective environment (36 out of 48 clones) and 1/8 of tobramycin selective environment (21 out of 48 clones) (see supplementary table 5, Supplementary Material online). In addition, the number of MIC declines within each treatment was dependent on the genetic background, being NfxB the genetic background presenting a higher percent of declines (59 out of 64 clones, *P* < 0.001), followed by MDR6 (44 out of 64 clones, *P* < 0.001) and ParR (42 out of 64 clones, *P* < 0.001). (*B*) The figure shows the degree of ceftazidime decline depending on the genetic background. The higher decline of ceftazidime MIC was observed in ParR (up to 128-fold), followed by NfxB (up to 11-fold) and MDR6 (up to 6-fold).

We then analyzed the relevance of both, the selective environment and the genetic background, in the decrease of ceftazidime MIC observed in that 76% of the clones. Nonselective environments (antibiotic-free and 1/16 of tobramycin MIC) were the ones in which a higher number of clones showed a decline of ceftazidime MIC (44 out of 48 clones), followed by the 1/4 of tobramycin MIC (36 out of 48 clones) and 1/8 of tobramycin MIC (21 out of 48 clones) selective environments (supplementary table 5, Supplementary Material online and fig. 4*A*). MIC changes were evaluated using log_2_ fold change with ANOVA followed by the corresponding post-hoc tests as described in Materials and Methods. The reduction of ceftazidime MICs observed within each environment was dependent on the genetic background, statistically ascertained both when considering number of changes and fold change (minimal support *P* < 0.005). In particular, the NfxB-CAZ genetic background presented the highest number of MIC declines (59 out of 64 clones), followed by MDR6-CAZ (44 out of 64 clones) and ParR-CAZ (42 out of 64 clones) (fig. 4*A*). Moreover, the genetic background also determined the degree of resistance decline (*P* < 0.001), being up to 128-fold in ParR (*μ* = 39.7), up to 11-fold in NfxB (*μ* = 2.65) and up to 6-fold in MDR6 (*μ* = 1.7) clones (fig. 4*B*). These data indicate that epistasis and the existence of different genetic backgrounds does not only constrain AR evolution (Hernando-Amado et al. 2019), but also compensatory evolution, something that had not been previously reported.

### Relative Fitness of ParR-CAZ-, NfxB-CAZ-, and MDR6-CAZ-Derived Clones

**Fig. 5.**
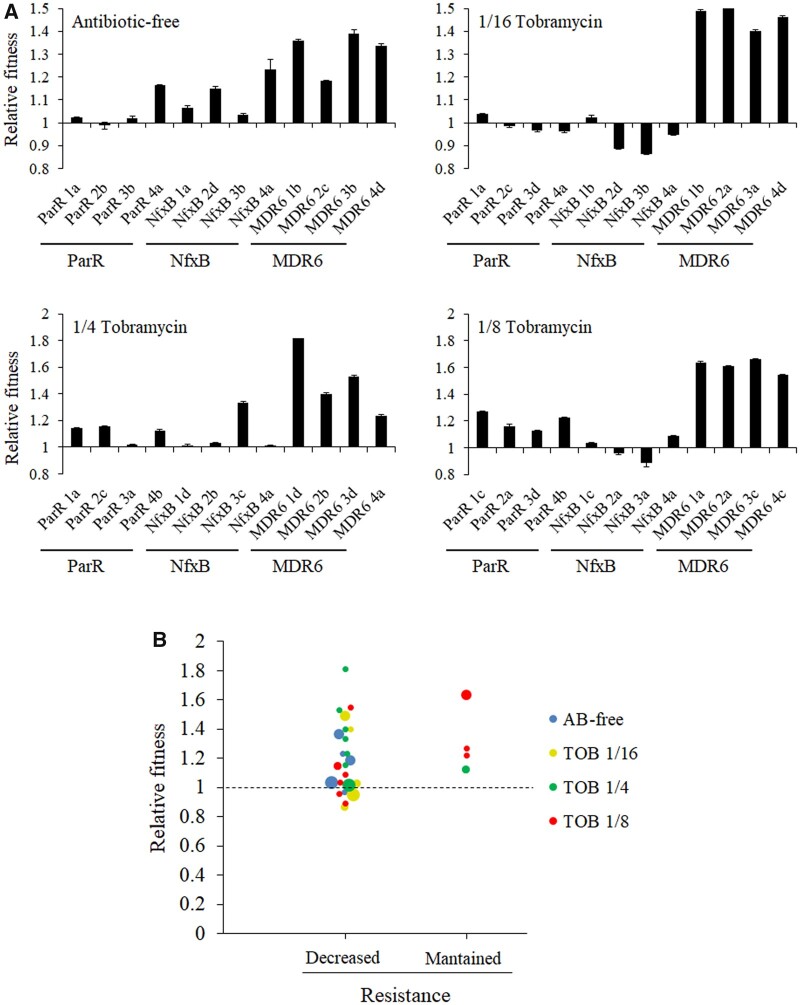
Impact of fitness recovery on AR decline. (*A*) Relative fitness of clones evolved 8 weeks in antibiotic-free and tobramycin sublethal environments. Growth curves of the resistant mutants, before and after antibiotic-free or tobramycin evolution, were recorded in antibiotic-free medium (LB). The fitness of each clone was measured as the area under the growth curve. Relative fitness of the clones was calculated respect to their parental strains. Relative fitness was increased (relative fitness value ≥1.1) in 67%, 33%, 67%, and 75% of clones isolated from populations evolved in antibiotic-free medium, 1/16 of tobramycin MIC, 1/4 of tobramycin MIC, and 1/8 of tobramycin MIC, respectively (see supplementary table 6, Supplementary Material online). The analysis also showed differences in fitness improvement within each genetic background. All the clones belonging to MDR6-CAZ presented an increased fitness followed by ParR-CAZ (50%) and NfxB-CAZ (31%). The mean of values from three replicates are represented. (*B*) Relationship between fitness and ceftazidime resistance decrease of clones evolved during 8 weeks in antibiotic-free and tobramycin sublethal environments. As shown, ceftazidime resistance decreases as a function of relative fitness in antibiotic-free and tobramycin sublethal environments. Each of the points represents a clone and the colors indicate different treatments. Relative fitness was increased (fitness value ≥1.1) in 58%, 33%, 60%, and 43% of clones that showed a resistance decrease after evolution in antibiotic-free, 1/16 of tobramycin MIC, 1/4 of tobramycin MIC, and 1/8 of tobramycin MIC, respectively (see supplementary table 6, Supplementary Material online). Importantly, relative fitness was also improved in all the clones presenting resistance maintenance. The size of the points is proportional to the number of clones within the same treatment presenting the same value.

We observed that decline of ceftazidime resistance can occur not only in antibiotic-free environments, where compensatory mutations may be a reasonable cause, but also in environments containing tobramycin, where tobramycin resistance mutations, which might also produce additional fitness costs that may influence the selection of said compensatory mutations, are expected to be acquired. Therefore, we compared fitness of the 48 ParR-CAZ-, NfxB-CAZ-, and MDR6-CAZ-derived clones with respect to their parental strains (ParR-CAZ, NfxB-CAZ, or MDR6-CAZ), by estimating the area under the growth curve recorded in antibiotic-free medium (fig. 5 and supplementary table 6, Supplementary Material online). Fitness of the selected clones evolved in antibiotic-free, 1/4 of tobramycin MIC, 1/8 of tobramycin MIC, and 1/16 of tobramycin MIC was improved in 67%, 67%, 75%, and 33% of them, respectively. After ANOVA, relative changes in fitness were tested with single-sample *t*-tests for *μ* = 1 (no change). An important fitness improvement was observed, as expected, in antibiotic-free medium (*P* = 0.001), where compensatory but no resistance mutations are expected. However, a high number of the clones evolved in 1/4 and 1/8 of tobramycin MIC environments, which may present mutations responsible for the increased tobramycin resistance, also showed an improvement of fitness (*P* = 0.0036 and *P* = 0.0033, respectively, fig. 5), suggesting that evolution toward improved fitness is uncoupled from tobramycin resistance evolution. ANOVA and Tukey also revealed major and significant differences in relative fitness across clones belonging to different genetic backgrounds (*P* < 0.001, fig. 5*A*). Namely, all the clones selected from MDR6-CAZ, the genetic background that originally showed the higher initial fitness cost (fig. 2), 50% of the clones belonging to ParR-CAZ and 31% of the clones belonging to NfxB-CAZ, showed an improved fitness after 8 weeks of ALE. This suggests that fitness improvement is influenced by the set of mutations initially present in parental strains. Although a further analysis would still be necessary, these results also suggest that the higher fitness cost associated with acquisition of resistance in a mutant, the greater the mutational space for compensatory evolution to occur, a question previously raised (Dunai et al. 2019) but that had not been analyzed until now.

We analyzed the possibility that resistance loss may be associated with an increase in fitness. We found both, an increase of relative fitness and a ceftazidime resistance decline in 58%, 60%, 43%, and 33% of the clones evolved in antibiotic-free, 1/4 of tobramycin MIC, 1/8 of tobramycin MIC, and 1/16 of tobramycin MIC, respectively; relative fitness was also improved in all clones that presented a maintenance of resistance (fig. 5*B* and supplementary table 6, Supplementary Material online). This indicates that the decline in the original ceftazidime resistance level may be, at least partly, associated with compensatory evolution of fitness costs, although a correlation between the level of fitness compensation and the level of ceftazidime resistance decline was not firmly established. It is worth noting here that tobramycin selects tobramycin resistant mutants, which may reduce the overall *P. aeruginosa* fitness, hence counteracting the fitness improvements associated with the decline of ceftazidime resistance.

### Decline of Ceftazidime Resistance Is Not Associated with Molecular Reversion of Resistance

ParR-CAZ-, NfxB-CAZ-, and MDR6-CAZ-derived clones were subjected to whole-genome sequencing, to gain insights into the underlying molecular causes of decline of ceftazidime resistance. Their genomes were compared with the ones of their parental strains, to determine new genetic events acquired during evolution. Some of the clones became mutators and, to facilitate the comparative analysis between replicates (within the same genetic background or across different genetic backgrounds), only those genes that also presented mutations in nonmutator clones were considered for this analysis. In the case of the clones evolved in antibiotic-free medium, a total of 59 genetic events were identified (supplementary table 7, Supplementary Material online), 15 in ParR-CAZ, 18 in NfxB-CAZ, and 26 in MDR6-CAZ genetic backgrounds. In the case of the clones evolved in 1/4 of tobramycin MIC, a total of 62 genetic events were identified (supplementary table 8, Supplementary Material online), 24 in ParR-CAZ, 18 in NfxB-CAZ, and 20 in MDR6-CAZ genetic backgrounds. In the case of the clones evolved in 1/8 of tobramycin MIC, a total of 46 genetic events were identified (supplementary table 9, Supplementary Material online), 22 in ParR-CAZ, 17 in NfxB-CAZ, and 7 in MDR6-CAZ genetic backgrounds. Finally, in the case of the clones evolved in 1/16 of tobramycin MIC, a total of 53 genetic events were identified (supplementary table 10, Supplementary Material online), 18 in ParR-CAZ, 24 in NfxB-CAZ, and 11 in MDR6-CAZ genetic backgrounds. The genome of the 48 clones was initially screened to determine whether molecular reversion of resistance (Durao et al. 2018) could account for the observed decline of ceftazidime resistance, although it is expected that phenotypic reversion, by the acquisition of new genetic variations, could be more likely than genetic reversion by the restoration of the mutated allele to a wild-type one (Levin et al. 2000; Dunai et al. 2019). The three mutants here analyzed present large deletions, a genetic event that cannot be restored, as well as mutations in different genes. None of the original mutations present in each of the parental genetic backgrounds reverted back to the wild-type sequence, so that compensatory evolution, and not genetic reversion, may be the cause of the observed decline of ceftazidime resistance.

### Genetic Variations Associated with the Acquisition of Tobramycin Resistance in Sublethal Tobramycin Environments

The genomes of 36 clones from the populations independently evolved during 8 weeks in different tobramycin sublethal concentrations, and presenting increased tobramycin resistance, were analyzed to gain insights into the underlying molecular causes of the acquisition of said resistance. It is relevant to remind that the original ceftazidime resistant mutants were tobramycin hyper-susceptible because they lack the aminoglycosides efflux pump *mexXY* (Hernando-Amado et al. 2020); their evolutionary trajectories toward tobramycin resistance could be different than those of the wild-type strain (Sanz-Garcia et al. 2018b). As mentioned above, a total of 62 genetic events were identified in the clones isolated from populations evolved in 1/4 of tobramycin MIC (supplementary table 8, Supplementary Material online), 46 genetic events were identified in the clones isolated from populations evolved in 1/8 of tobramycin MIC (supplementary table 9, Supplementary Material online), and a total of 53 genetic events were identified in the clones isolated from populations evolved in 1/16 of tobramycin MIC (supplementary table 10, Supplementary Material online), although some of these variations were most likely compensatory genetic events acquired during evolution (supplementary table 11, Supplementary Material online). Variations in a limited number of genes, 6 in total, were commonly acquired in different sublethal tobramycin environments and genetic backgrounds (supplementary table 12, Supplementary Material online). Among them, genetic variations in *fusA*, encoding an elongation factor whose mutations are clearly associated with acquisition of aminoglycoside resistance in clinical and nonclinical strains (Feng et al. 2016; Bolard et al. 2018; Ibacache-Quiroga et al. 2018; Lopez-Causape et al. 2018; Sanz-Garcia et al. 2018b), were commonly selected in the three genetic backgrounds analyzed. In particular, variations in *fusA* were detected in all ParR-CAZ- and NfxB-CAZ-derived clones and some MDR6-CAZ-derived clones (MDR6 1d and 4a clones), isolated from populations evolved in 1/4 of tobramycin MIC (supplementary table 8, Supplementary Material online), and in all NfxB-CAZ-derived clones isolated from populations evolved in 1/8 of tobramycin MIC (supplementary table 9, Supplementary Material online). In addition, mutations in *ptsP*, encoding a phosphoenolpyruvate phosphotransferase, whose mutation is associated with acquisition of tobramycin resistance (Schurek et al. 2008; Sanz-Garcia et al. 2018b), were acquired in ParR-CAZ and MDR6-CAZ genetic backgrounds, whereas they were not selected in the NfxB-CAZ genetic background. In particular, mutations in this gene were acquired in clones ParR 1a, 3a, and 4b and in clone MDR6 3d, isolated from populations evolved in 1/4 of tobramycin MIC (supplementary table 8, Supplementary Material online) and in all ParR-CAZ-derived clones, isolated from populations evolved in 1/8 of tobramycin MIC (supplementary table 9, Supplementary Material online). The fact that all the ParR-CAZ-derived clones, and no NfxB-CAZ-derived clones, isolated from populations evolved in 1/8 of the MIC of tobramycin present mutations in *ptsP*, and that all NfxB-CAZ-derived clones, and no ParR-CAZ-derived clones, isolated from populations evolved in 1/8 of the MIC of tobramycin present mutations in *fusA*, illustrates to which extent the genetic background can constrain the evolution of AR (Hernando-Amado et al. 2019; Hernando-Amado et al. 2020; Card et al. 2021).

### Intragenic Compensatory Mutations in the AmpR Regulator and the RpoB Subunit of the RNA Polymerase

The genome of the 12 clones evolved during 8 weeks in antibiotic-free medium was analyzed to identify all possible compensatory genetic events acquired during evolution (supplementary table 7, Supplementary Material online). Afterwards, genetic variations in these genes were searched within those identified in the clones evolved during 8 weeks in sublethal tobramycin environments (supplementary tables 8–10, Supplementary Material online) to distinguish between compensatory mutations and genetic events leading to tobramycin resistance. Variations in a limited number of genes, 9 in total, were commonly acquired in both, antibiotic-free and sublethal tobramycin environments (supplementary table 11, Supplementary Material online). Among them, some mutations were located in genes that were originally mutated in the parental genetic backgrounds ParR-CAZ and MDR6-CAZ (supplementary table 13, Supplementary Material online), supporting that intragenic compensation might be the basis for alleviating the fitness costs associated with acquisition of ceftazidime resistance in these mutants. Indeed, in ParR-CAZ-derived clones we observed intragenic mutations in *ampR*, which encodes an LTTR that regulates the expression of the β-lactamase AmpC (Kong et al. 2005; Alvarez-Ortega et al. 2010; Balcewich et al. 2010; Gifford et al. 2018; Sanz-Garcia et al. 2018a), and in MDR6-CAZ-derived clones, in *rpoB*, which encodes the subunit β of the RNAP (Villain-Guillot et al. 2007), whose mutation frequently confers rifampicin resistance (Severinov et al. 1993; Qi et al. 2014) (supplementary table 13, Supplementary Material online).

**Fig. 6.**
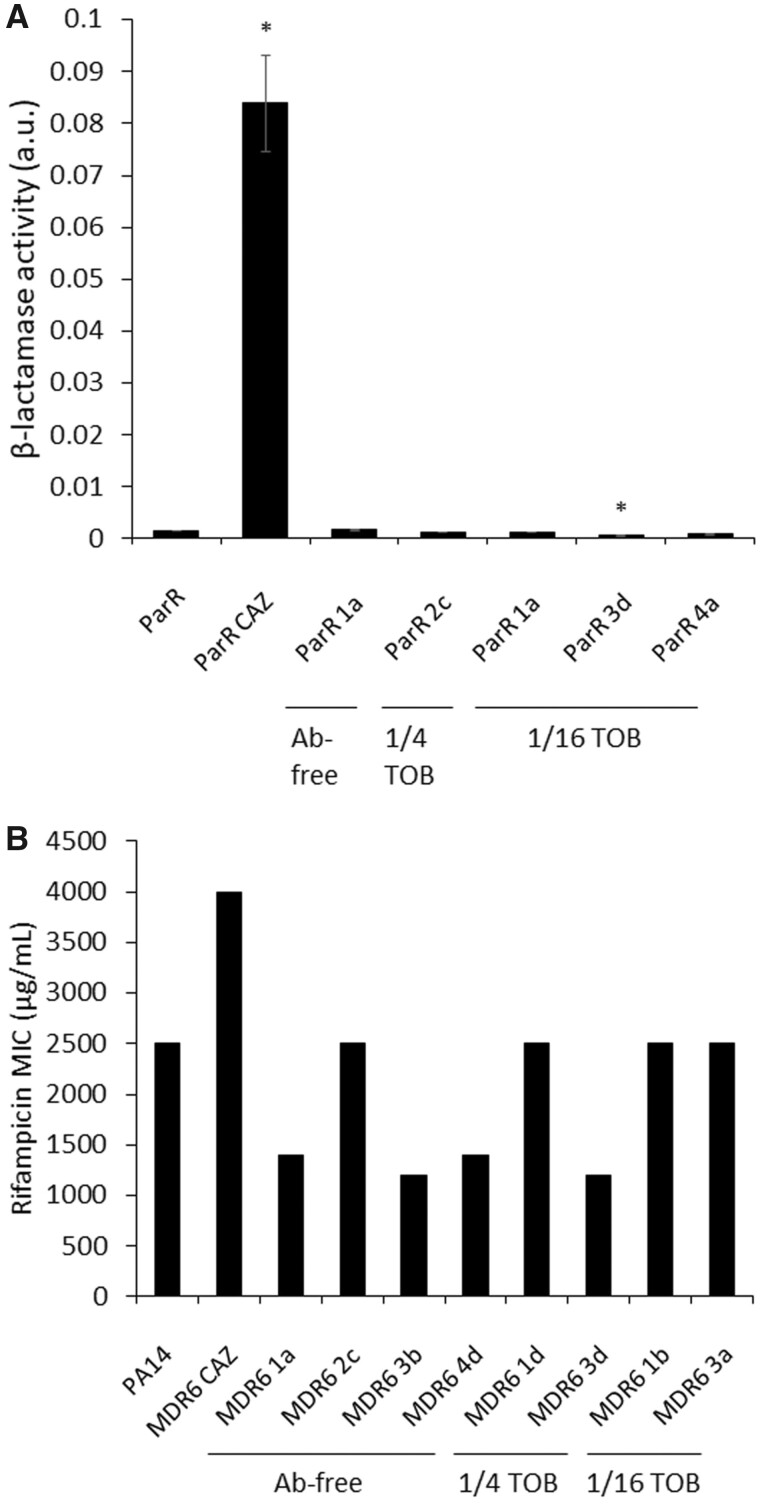
Reversion of phenotypes due to intragenic secondary mutations in *ampR* and *rpoB*. (*A*) Figure shows β-lactamase activity of protein extracts from ParR, ParR-CAZ, and Par-CAZ-derived clones: ParR 1a, isolated from a population evolved in antibiotic-free medium, ParR 2c isolated from a population evolved in 1/4 of tobramycin, or ParR 1a, 3d, and 4a isolated from populations evolved in 1/16 of tobramycin MIC sublethal concentration. β-Lactamase activity was increased up to 55-fold in ParR-CAZ mutant respect to its parental strain, whereas a reversion of this phenotype was achieved after 56 days of ALE in antibiotic-free and tobramycin sublethal environments and the acquisition of secondary mutations in *ampR*. Even a significantly reduced β-lactamase activity respect to the original ParR parental strain was measured for one of them. Error bars indicate standard deviations of the results from three independent biological replicates. Statistically significant differences regarding ParR were calculated with *t-*test for paired samples assuming equal variances: *P < 0.05. (*B*) Rifampicin MIC (μg/ml) of PA14 wild-type strain, MDR6-CAZ parental strain, MDR6-CAZ-derived clones: MDR6 1b, 2c, 3b, and 4d isolated from populations evolved in antibiotic-free medium, clones MDR6 1d and 3d isolated from populations evolved in 1/4 of tobramycin MIC sublethal concentration, and clones MDR6 1b and 3a isolated from populations evolved in 1/16 of tobramycin MIC sublethal concentration are represented. As shown, MDR6-CAZ parental strain has an increased rifampicin resistance respect to PA14 wild-type strain, whereas a reversion of this phenotype was achieved after 56 days of ALE in antibiotic-free and tobramycin sublethal environments and the acquisition of secondary mutations in *rpoB*.

The ParR-CAZ genetic background, originally presented an amino acid variation in AmpR (Asp135Asn) (supplementary table 1, Supplementary Material online), and displayed intragenic mutations in *ampR* after 8 weeks of evolution in both, antibiotic-free medium and tobramycin sublethal concentrations. Clone ParR 1a, isolated from a population evolved in antibiotic-free medium, presented a Pro164Leu amino acid variation, clone ParR 2c, isolated from a population evolved in 1/4 of tobramycin MIC, presented an His39Tyr amino acid variation, and clones ParR 1a, 3d, and 4a, isolated from populations evolved in 1/16 of tobramycin MIC sublethal concentration, presented two distinct Glu274fs and Leu110Pro amino acid modifications, respectively (supplementary table 13, Supplementary Material online). In addition, clone ParR 3 b isolated from a population evolved in antibiotic-free medium presented a Phe19fs amino acid variation in AmpC. AmpR is a transcriptional regulator that controls the expression of *ampC* (Balcewich et al. 2010) and, therefore, the level of resistance to β-lactam antibiotics. The ParR-CAZ parental genetic background, that presents an amino acid variation in AmpR (Asp135Asn) (supplementary table 1, Supplementary Material online), is highly resistant to ceftazidime (MIC > 256 μg/ml; supplementary table 3, Supplementary Material online) showing an increased β-lactamase activity (up to 55-fold) with respect to its ParR parental strain (fig. 6*A*). In addition, the ParR-CAZ mutant presents a high fitness cost that could be partly associated with the mutation present in *ampR* (Perez-Gallego et al. 2016). We hypothesize that the intragenic mutations in *ampR* acquired after 8 weeks of ALE in antibiotic-free and sublethal tobramycin concentrations could modify AmpR activity, reducing the amount of AmpC and, therefore, β-lactamase activity. Actually, a strong decline of ceftazidime resistance (up to 128-fold) was observed in ParR-CAZ-derived clones. In particular, clone ParR 1a, isolated from populations evolved in antibiotic-free medium, ParR 2c isolated from a population evolved in 1/4 of tobramycin sublethal concentration, or ParR 1a, 3d, and 4a, isolated from populations evolved in 1/16 of tobramycin MIC sublethal concentration, presented a reduced ceftazidime MIC of 128-fold, 32-fold, or 64-, 85-, and 43-fold, respectively, compared with the ParR-CAZ parental strain (supplementary table 5, Supplementary Material online). Confirming this hypothesis, a reduced β-lactamase activity (up to 140-fold) was observed in these compensated, evolved clones (fig. 6*A*), indicating that the intragenic mutations acquired in *ampR* (supplementary table 13, Supplementary Material online) are the main cause of ceftazidime resistance decline in ParR-CAZ genetic background.

**Fig. 7.**
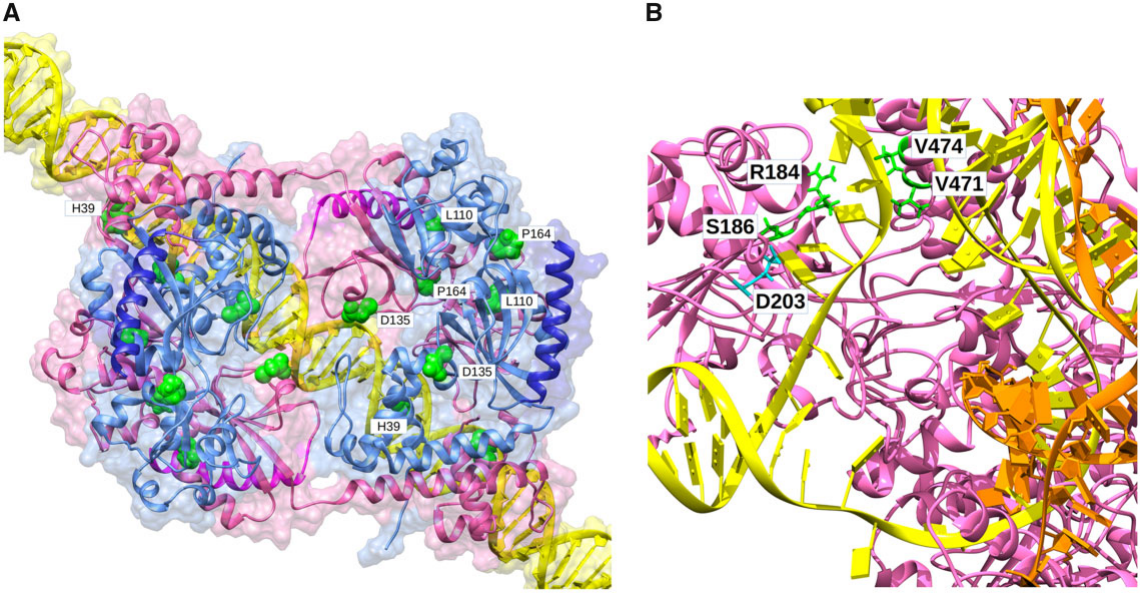
Modeling of the effect of AR and compensatory mutations in the structures of AmpR and RpoB. (*A*) Proposed model of the AmpR tetramer bound to the putative promoter of *ampC* of *P. aeruginosa* PA14. Mutated amino acids are shown as balls for better identification. Only amino acids in one pair of monomers have been labeled. The C-terminal regions affected by 274 frame shifts are highlighted. D135N and H39Y would affect promoter recognition, F19fs truncates the protein, L110P would destabilize the complex, P164L would destabilize the effector binding pocket, and G274fs would affect interaction with RNAP. (*B*) Proposed model of RpoB bound to a DNA bubble and a nascent RNA molecule. Mutated amino acid D203, whose mutation causes the resistant phenotype (possibly through a change in promoter specificity), and amino acids with reversion mutations are shown as sticks. D203E would affect the interaction with the open DNA bubble, pushing the nontranscribed (NT) strand toward the channel between R184 and V471-V474. Reversion mutants S186F, R184C, V471E, and V474E would correct stacking, tensions, and “scrunching” distortions in the NT strand.

In order to interpret the effect of *ampR* mutations, the AmpR structures of the wild-type, ceftazidime resistant and ceftazidime compensated mutants were modeled. Structure modeling of the different AmpR mutants in a DNA-bound tetramer complex formed by two dimers composed of a compact and extended form of AmpR each and bound to DNA, sheds new light on the observed resistance phenotype (fig. 7*A*). The Asp135Asn mutation, present in the mutant ParR-CAZ is located in a region involved in DNA binding, where this new amino acid likely stabilizes the AmpR binding to the intergenic region that contains the *ampC* and *ampR* promoters, leading to constitutive expression of *ampC*. For its part, the various reversal phenotypes can be explained by changes that hamper AmpR function: Phe19fs results in a truncated, defective protein, His39Tyr lies in a DNA-binding helix likely reducing affinity for the promoter and hence expression of *ampC*; Leu110Pro would affect one of the helices in the intermonomer interface destabilizing packing and formation of the active complex; Pro164Leu is located at the hinge between the two domains that line the effector-binding pocket, likely rendering AmpR unresponsive to its activators, and Glu274 frame shifts would alter the C-terminal domain, impairing the interaction with RNAP and therefore transcription of genes regulated by AmpR (Balcewich et al. 2010).

The MDR6-CAZ genetic background, originally presenting an amino acid variation in RpoB (Asp203Glu) (supplementary table 1, Supplementary Material online), suffered intragenic mutations in *rpoB* after 8 weeks of evolution in both, antibiotic-free and sublethal tobramycin environments. Clones MDR6 1b, 2c, 3b, and 4d, isolated from populations evolved in antibiotic-free medium, presented an Arg184His, Arg184Cys, Ser186Phe, and Val474Glu amino acid variation, respectively (supplementary table 13, Supplementary Material online). Clones MDR6 1d and 3d, isolated from populations evolved in 1/4 of tobramycin MIC sublethal concentration, showed a Ser186Phe amino acid modification, and clones MDR6 1b and 3a, isolated from populations evolved in 1/16 of tobramycin MIC sublethal concentration, presented a Val471Glu and Arg184Cys amino acid modification, respectively (supplementary table 13, Supplementary Material online). It is well-known that *rpoB* mutants present pleiotropic effects, including rifampicin resistance (an antibiotic that targets RpoB), reduced transcriptional efficiency, altered expression of essential genes, or DNA breaks (Severinov et al. 1993; Qi et al. 2014). Strikingly, the ceftazidime resistant MDR6-CAZ parental strain (supplementary table 5, Supplementary Material online), presenting an Asp203Glu variation in RpoB, displays a high fitness cost in antibiotic-free medium (fig. 2) and, in addition to resistance to ceftazidime, it also presents resistance to rifampicin (MIC > 4 mg/ml; fig. 6*B*), supporting that RpoB function is affected in this mutant. We hypothesize that the intragenic mutations acquired in *rpoB* after 8 weeks of ALE in antibiotic-free and sublethal tobramycin concentrations could be mutations that compensate fitness through restoring RpoB functionality. Indeed, secondary mutations in *rpoB* have been described to restore the defective RNAP activity caused by mutations in this gene in *P. aeruginosa* (Hall et al. 2010; Hall and MacLean 2011). All the mentioned MDR6-CAZ-derived clones presented an increased fitness, measured as growth in antibiotic-free medium (fig. 5*A*). In addition, rifampicin and ceftazidime MIC of the mentioned clones were reduced up to 3.3- and 6-fold, respectively (fig. 6*B* and supplementary table 5, Supplementary Material online). The reduction of rifampicin MIC suggests that the secondary *rpoB* mutation allows RpoB to recover functionality close to that of the wild-type.

The effect of the *rpoB* intragenic mutations in the activity of the RNAP can be explained by carefully analyzing the predicted models of RpoB substituted into a hybrid *Escherichia coli* RNAP complex with DNA bubbles and nascent RNAs (fig. 7*B*). Asp203 is followed by two arginines and is spatially close to Ser186. These amino acids interact with the beginning of the transcription bubble (Zhang et al. 2012) in all reported complexes, likely helping to open the DNA bubble in a zipper-like fashion, and contributing to reorient nucleotide bases in the nontranscribed (NT) DNA strand away from the transcribed (T) strand. Asp203Glu introduces a large side chain and a negative charge that force a reorientation of the arginines and a slight displacement of the NT strand. We propose that, at such an early point in the bubble, the small displacement of the NT strand also has a small effect in the nearby T strand; this would not be enough to affect interactions with the active site and transcription, but as the distortion is transmitted through the T strand, it would allow the nascent RNA to grow beyond the third nucleotide stage. In addition, Asp203Glu would affect the “scrunching” of the DNA bubble, pushing it in the cavity toward a channel open between Arg184 and Val471-Val474, whose interaction with the sixth, seventh, and eighth nucleotides of the bubble’s NT strand might modify its tension, possibly affecting the progression of transcription. The question remains how these structural changes are associated with resistance to ceftazidime of this mutant. The fact that *rpoB* mutations, besides rifampicin resistance, can select pleiotropic mutants presenting resistance to other drugs, as quinolones and ampicillin (Pietsch et al. 2017), suggests that transcriptional changes associated with RpoB malfunctioning might be the underlying molecular cause of ceftazidime resistance in these mutants. Concerning the effect of compensatory mutations on RpoB functionality, Ser186Phe introduces a large side chain close to Asp203Glu that would rearrange the stacking of the first bases in the NT strand correcting the changes of the latter amino acid change. The effect of Arg184His and Arg184Cys could be explained by the reduction in side chain size, which would leave more space and relieve the tension on the NT strand. Finally, the effect of Val471Glu and Val474Glu might be explained by the introduction of a repulsive charge and larger side chain pushing the NT strand toward Arg184 at the opposite wall, hampering progression of the NT strand through the channel between them and Arg184 and constraining release of the tension in the DNA bubble. Thus, the seemingly most plausible explanation is that the intragenic mutations in *rpoB* would compensate the distortion introduced by the amino acid change Asp203Glu, restoring the orientation of the T strand. We hypothesized that these structural changes may be responsible for restoring RpoB functionality, hence allowing the recovery of fitness and the decline of ceftazidime resistance in the analyzed clones presenting these compensatory mutations.

The above-mentioned intragenic modifications acquired in *ampR* and *rpoB*, both in antibiotic-free and in sublethal tobramycin environments but specifically in ParR-CAZ and MDR6-CAZ genetic backgrounds, respectively, which originally presented mutations in these genes, shows the importance of genetic background in constraining compensatory evolution.

## Discussion

Recent years have seen a growing interest in the development of evolutionary approaches to tackle AR from a conservative point of view, improving the use of available antibiotics (Laxminarayan 2014). In this sense, numerous studies have proposed to exploit some evolutionary trade-offs associated with the acquisition of AR, as CS (Imamovic and Sommer 2013; Kim et al. 2014; Pal et al. 2015; Baym et al. 2016; Barbosa et al. 2018; Imamovic et al. 2018; Barbosa et al. 2019; Dunai et al. 2019; Hernando-Amado et al. 2020) or the possible decline of AR in antibiotic-free environments (Dunai et al. 2019).

The evolutionary forces that modulate the emergence of a specific AR mutation have been studied in detail; mutation rate and mutational input, clonal interference, cross-selection, level of resistance, fitness under selection, epistatic interactions, and contingency are factors that constrain the evolutionary trajectories leading to AR (Weinreich 2005; de Visser and Krug 2014; Szamecz et al. 2014; Maddamsetti et al. 2015; Gifford et al. 2016; Hughes and Andersson 2017; Imamovic et al. 2018; Nichol et al. 2019; Rosenkilde et al. 2019). Fixation of such mutations requires that they persist, even when selective pressure is suspended. For this stability to happen, fitness costs associated with the acquisition of AR should be low or easily compensated by the acquisition of secondary mutations that do not reduce AR to the selective antibiotic (Bjorkman, Nagaev et al. 2000; Nagaev et al. 2001; Marcusson et al. 2009). Although some detailed studies on the effect of fitness costs in the stability of AR have been published, most works still focus on the emergence of AR. Information on the degree to which compensatory evolution is contingent on genetic background, on initial fitness costs and/or on the environmental conditions and how these factors impact the stability or reversion of AR, as well as their associated trade-offs (i.e., CS), is almost absent in the field.

It is debatable whether reversion of resistance upon antibiotic therapy discontinuation is a common situation (Schrag et al. 1997; Enne et al. 2001; Andersson and Hughes 2010; Brolund et al. 2010), because it has been shown that eliminating the use of an antibiotic in a geographical area does not revert resistance (Sundqvist et al. 2010). Actually, decline of resistance is not a black-and-white issue, but rather depends on different factors. An important one is the type of antibiotic used to treat an infection (Seppala et al. 1997; Enne et al. 2001; Gottesman et al. 2009; Andersson and Hughes 2010; Dunai et al. 2019), and hence, the selected resistance mechanisms, because the fitness cost associated with acquiring resistance depends on the cellular functions affected (Baquero et al. 2009; Melnyk et al. 2015). Consequently, the identification of antibiotics for which resistance could be unstable in absence of selection is of utmost relevance for the design of evolution-based approaches to tackle AR. Here, we have identified compensatory mutations responsible for the decline of ceftazidime resistance that, importantly, do not affect pre-existing CS to tobramycin.

CS has been explored as a trade-off potentially exploitable to tackle AR, besides fitness costs associated with the AR acquisition. For that, CS must be robust and emerge in different genetic backgrounds, being particularly relevant the case of pre-existing antibiotic resistant mutants (Hernando-Amado et al. 2020), because infections caused by *P*. *aeruginosa* in CF patients can contain different strains with distinct antibiotic susceptibility profiles (Lopez-Causape et al. 2017). In addition, CS must be stable. The stability of CS, which had not been explored, is particularly relevant for the implementation of antibiotic use policies, such as evolutionary-based therapies alternating two different antibiotics, using the antibiotic for which there is CS. Here, we have observed that compensatory evolution of fitness costs associated with ceftazidime resistance in antibiotic-free environments does not affect pre-existing CS to tobramycin.

We observed a robust decline of ceftazidime resistance, within 450 generations, in 92% of the clones isolated from populations evolved during 8 weeks in antibiotic-free medium as well as in populations evolved in sublethal concentrations of tobramycin. These results agree with a clinical study at a Shanghai hospital, in which the restriction of ceftazidime consumption resulted in a significant decrease of ceftazidime resistance levels in *P. aeruginosa* (Guo et al. 2015). In addition, we found that the degree and frequency of ceftazidime resistance decline is dependent on the genetic background, showing ParR-CAZ-derived clones the highest decrease and NfxB-CAZ-derived clones the most frequent decline. As far as we know, this is the first work describing that decline of AR is contingent on genetic background and that it can occur in environments containing sublethal antibiotic concentrations. Further, it is important noticing that, in addition, we observed that compensatory evolution in antibiotic-free medium allows the recovery of *P. aeruginosa* susceptibility to drugs to which these mutants presented cross-resistance (other β-lactams).

Not only the type but the strength of selection (i.e., lethal or sublethal antibiotic concentrations) can restrict the type of AR mutations acquired (Sanz-Garcia et al. 2020) and their associated fitness costs. In this work, we answer the question of the extent to which sublethal concentrations of tobramycin that can be found in certain clinical situations (Baquero and Negri 1997) may determine, beyond the evolution of tobramycin resistance either the evolution of CS to ceftazidime or compensatory evolution (and a possible decline of ceftazidime resistance), allowing a switch back to this drug. Sublethal tobramycin concentrations (1/4 and 1/8 of tobramycin MIC) selected tobramycin resistant mutants in the three analyzed genetic backgrounds, which is in agreement with the described sublethal selective window for tobramycin in *P. aeruginosa* PA14 (Sanz-Garcia et al. 2020). However, tobramycin resistance was not only selected in the presence of 1/4 and 1/8 of tobramycin MIC but also in the presence of 1/16 of tobramycin MIC in the MDR6-CAZ and some subpopulations of the ParR-CAZ genetic backgrounds. This indicates that the probability to select resistance at a particular sublethal tobramycin concentration depends on the starting genetic background, a feature that had not been previously explored. In addition, we observed that ceftazidime resistance declines in these sublethal tobramycin concentrations due to compensatory evolution.

From a genetic point of view, reversion or decline of AR can result from a molecular (Durao et al. 2018) or phenotypic reversion of resistance (Levin et al. 2000; Dunai et al. 2019), by the restoration of the mutated allele to a wild-type one or the acquisition of new genetic variations, respectively. In this work, after whole-genome sequencing of 48 clones presenting a decline of ceftazidime resistance, the first possibility was discarded, because none of the original mutations present in each of the parental genetic backgrounds reverted back to the wild-type sequence. In fact, intragenic compensatory mutations in *ampR* and *rpoB*, specifically acquired in ParR-CAZ- and MDR6-CAZ-derived clones, respectively, seem to be responsible for the decline of ceftazidime resistance observed in these mutants.

Besides their relevance in the AR field, our results provide general information for the theory of evolution. The evolution of *P. aeruginosa* in the presence of ceftazidime leads to three phenotypes of interest from the clinical perspective: ceftazidime resistance is adaptive to the selective pressure whereas their associated trade-offs, fitness costs and CS to tobramycin, are not. The evolutionary process is usually considered to increase complexity by the cumulatively acquisition of adaptive genetic changes, giving evolution a direction, an arrow of time (Ekstig 2015). Our results support that this arrow only operates if the adaptive phenotype (i.e., ceftazidime resistance) is preserved -fixed- when selection ends; otherwise, processes of short-sighted evolution could be expected (Levin and Bull 1994; Martinez 2013). Although here we found that resistance to ceftazidime declines in absence of selection, and hence it is not fixed, we also observed that its evolutionary trade-off, CS to tobramycin, remains. This implies that stable, not necessarily adaptive phenotypes might have a relevance in evolution that is currently underestimated.

Antibiotic cycling has been usually unsuccessful in reducing AR burden (Schuetz and Beardmore 2018; van Duijn et al. 2018). One of the underlying reasons could be that, when implemented, these programs have been mainly based on blind assays, under the assumption that any AR mechanism would present a fitness cost and supposing that AR would be lost under exposure to the subsequent antibiotic (Nichol et al. 2018). However, for decline of AR to occur, the adaptation to a specific drug must cause the emergence of conserved trade-offs, such as high fitness costs in antibiotic-free environments or CS to a second drug. Further, compensatory evolution of fitness costs does not unequivocally imply a decline of AR, because it may depend on the antibiotic and the resistance mutations involved (Seppala et al. 1997; Enne et al. 2001; Gottesman et al. 2009; Andersson and Hughes 2010; Dunai et al. 2019) and on other factors, as the ones here described: genetic background, initial fitness costs and strength of selection.

**Fig. 8.**
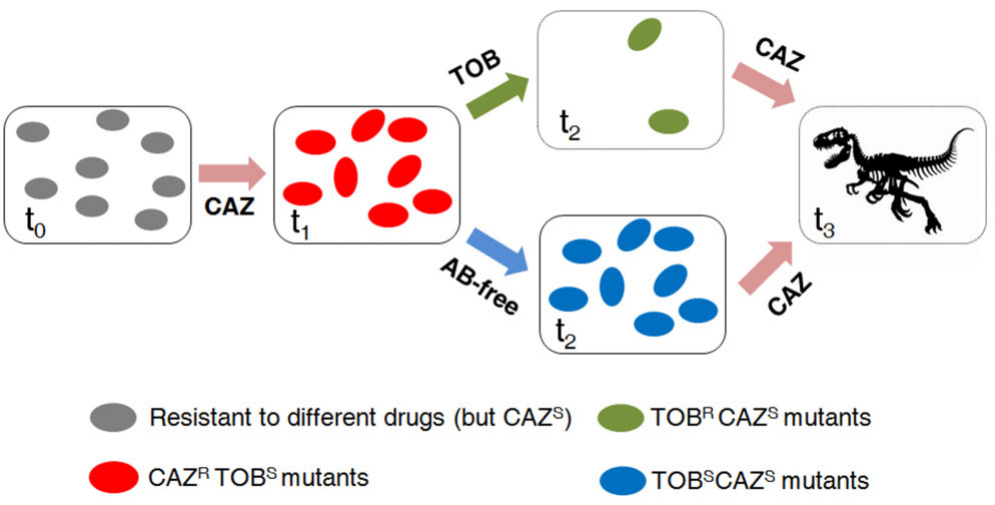
Conceptual figure illustrating two different evolutionary approaches to drive different antibiotic resistant mutants of *P. aeruginosa* to extinction. Different genetic backgrounds of *P. aeruginosa* (*t*_0_) show a robust evolution toward CS to tobramycin after short-term evolution in the presence of ceftazidime (*t*_1_) (Hernando-Amado et al. 2020). Furthermore, these CAZ^R^TOB^S^ mutants also have a high fitness cost in antibiotic-free environments. Therefore, it is possible to alternate the use of ceftazidime with periods of drug restriction, allowing the compensation of fitness costs and the decline of ceftazidime resistance (*t*_2_), or with tobramycin (Hernando-Amado et al. 2020), driving the populations to extinction (*t*_2_). In the second case, the hypothetical presence of sublethal tobramycin concentrations in certain situations, although selecting an increase of tobramycin resistance, would also lead to a decline of ceftazidime resistance in these populations. Therefore, a switch back to ceftazidime in either of the two strategies could finally result in the extinction of the resistant populations (*t*_3_).

Here we propose that the alternation of ceftazidime with tobramycin for the treatment of *P. aeruginosa* infections, even those due to pre-existing antibiotic resistant mutants, could be followed by a switch back to ceftazidime, to drive tobramycin resistant mutants that could result from sublethal tobramycin concentrations present in certain tissues or situations to extinction. In addition, we suggest that it might be possible to alternate the use of ceftazidime with periods of drug restriction, allowing a rapid decrease of ceftazidime resistance, preserving tobramycin CS (see conceptual fig. 8). However, it is important to emphasize that our results should not be generalized to other antibiotics or specific situations. Specific ALE assays, as those done here, are necessary to find drugs for which periods of drug restriction or drug-cycling policies could be effective.

## Materials and Methods

### Growth Conditions and Antibiotic Susceptibility Assays

Unless stated otherwise, bacteria were grown in Luria Bertani (LB) Broth at 37 °C and shaking of 250 rpm, in glass tubes. Antibiotic susceptibility was determined for ceftazidime, tobramycin, aztreonam, imipenem, tetracycline, fosfomycin, ciprofloxacin, and chloramphenicol at 37 °C, in Mueller Hinton (MH) agar, using Test strips (MIC Test Strip, Liofilchem). Rifampicin susceptibility (above the limit of detection of E-test strips) was determined by broth microdilution method in 96-well plates with round bottoms (Thermo Scientific Nunc) filled with MH supplemented with growing concentrations of antibiotic and bacteria at initial OD_600 nm_ of 0.01, after incubation at 37 °C for 24 h.

### Adaptive Laboratory Evolution Experiments

Three ceftazidime resistant mutants of *P. aeruginosa* PA14—four replicates each—were subjected to 8 weeks of ALE in presence or absence of sublethal concentrations of tobramycin (1/4, 1/8, and 1/16 of MIC of each linage), resulting in a total of 48 independent bacterial populations (12 grown in presence of 1/4 of tobramycin MIC, 12 in 1/8 of tobramycin MIC, 12 in 1/16 of tobramycin MIC, and 12 grown without antibiotic). Cultures were grown at 37 °C and 250 rpm for 56 days. Every day, the cultures were diluted (1/100), adding 10 µl of bacteria in 1 ml of fresh LB, either containing or lacking tobramycin. Each tobramycin concentration was maintained during ALE. Every replicate population was preserved, at the end of the experimental evolution, at −80 °C. In addition, the MIC of the antibiotic used for selection in populations (tobramycin) and of the one to which the mutants were initially resistant (ceftazidime), was determined at 37 °C in MH agar, using E-test strips.

### Growth Measurements

Growth curves were obtained inoculating overnight cultures to a final OD_600 nm_ of 0.01 in LB, by triplicate, in a 96-well microtiter plate (Nunclon Delta Surface) and by measuring the OD_600 nm_ of the bacterial cultures every 10 min during 20 h at 37 °C using a Spark 10M Plate Reader (Tecan). Fitness (W) was measured as the area under the growth curve recorded in antibiotic-free medium. Fitness cost of each ceftazidime resistant mutants respect to its parental strain was calculated as stated (Dunai et al. 2019), using the equation: 1 − (*W*_mutant_/*W*_parental strain_) and was expressed as percentage.

### Whole-Genome Sequencing and Analysis

The genomic DNA of each isolated clone was extracted using the Gnome DNA kit (MP Biomedicals). Whole-genome sequencing (WGS) and DNA quality was performed by Macrogen. Pair-end libraries (2 × 150) were constructed with Truseq DNA PCR free and sequenced using an Illumina NovaSeq6000 system. Coverage was greater than 300× for all samples. Genome sequence, gene coordinates, and annotations were obtained from GenBank nucleotide database. The quality of Illumina short reads was verified using FASTQC (Wingett and Andrews 2018). Reads were aligned against *P. aeruginosa* genome UCBPP-PA14 (NC_008463.1) using RNA-STAR (Dobin et al. 2013). Optical and PCR duplicates were marked using MarkDuplicates (Picard) function of The Genome Analysis Toolkit (McKenna et al. 2010). Alignment files in BAM format were indexed using SAMtools (Li et al. 2009). SNPs and small insertions/deletions (INDELs) were detected using freebayes (Garrison and Marth 2012). Only primary alignments were considered as effective reads. Impact of detected SNP and INDEL was evaluated using SnpEff (Cingolani et al. 2012) and annotated results were saved in VCF format. Genomic regions with no coverage (large chromosomal deletions) were identified using BEDTools (Quinlan and Hall 2010). Relevant genetic variants were detected with the help of SNPer viewer (https://bioinfogp.cnb.csic.es/tools/snper) and IGV browser (Thorvaldsdottir et al. 2013).

### β-Lactamase Activity Determination

Cells from overnight 20 ml cultures were harvested by centrifugation at 7,000 rpm for 10 min and then suspended in 500 μl of 0.1 M Na_2_HPO_4_ (pH 7.4) buffer. Crude protein extracts were obtained after sonication at 0.7 Hz of the suspended cells and subsequent centrifugation for 15 min at 13,000 rpm. Bradford protein assay with bovine serum albumin was performed for determining the protein content of each extract. Nitrocefin (Oxoid) was added to an equal amount of protein from each extract at a final concentration of 500 μg/ml, with Na_2_HPO_4_ 0.1M (pH 7.4) as the test buffer. The β-lactamase activity was quantified by measuring OD_486 nm_ every 2 min during 2 h at 37 °C in an Infinite M200 plate reader (Tecan).

### Statistical Analysis

R package AMR (Berends et al. 2021) was used to check all MIC values against EUCAST for classification of susceptibility. Collected MIC data were analyzed with R ((Version 4.0), retrieved from https://cran.r-project.org) using whole data sets and subsets by treatment, genetic background and their combinations: Multi- and One-Way ANOVA tests applying Welch’s correction for unequal variances, were used to test each and all variables and their combinations. Successful ANOVA was followed by post-hoc Tukey’s HSD all versus all tests. As Tukey may be too demanding for many of the desired comparisons, MIC changes over time were subject to longitudinal analysis using the parental strain values as time-zero reference: endpoint analyses relied on paired *t*-tests, and time series analyses used Dunnett’s test with Hochberg correction (Hochberg 1988), which was also used for other comparisons of multiple groups against a single reference.

Changes in susceptibility to various antibiotics were asserted with ANOVA and a two-sample test for equality of proportions of resistant and susceptible populations. The significance of log_2_ fold changes was tested using ANOVA, Welch *F*-Test and post-hoc Tukey HSD tests. Significance of relative changes in fitness was checked, after ANOVA, using single-sample *t*-tests with *μ* = 1 (no change). Kruskal–Wallis and Wilcoxon tests were used for count data. Comparisons between punctual MIC reference values of representative clones were calculated on a spreadsheet determining the 95% confidence interval of the mean of the log-transformed replicated experimental data and testing the log-transformed reference for inclusion in the computed interval (or the reference value in the back-transformed interval; Turnidge et al. 2006).

### Molecular Modeling

Models of wild-type and mutants of AmpR were obtained with I-TASSER (Yang et al. 2015) and Modeller (Webb and Sali 2016) using the corresponding sequences for *P. aeruginosa* UCBPP-PA14. Separate models for the extended and compact conformations of wild-type and mutant variants of AmpR were obtained using different combinations of reference structures (PDB: 5MMH, 4WKM, 3FZV, 3T1B). The structures were inspected to choose representative models using UCSF Chimera (Pettersen et al. 2004). A tetramer complex composed of two homodimers of AmpR, each with one subunit in extended and one in compact conformation, attached to the PA14 *ampC*-*ampR* intergenic region DNA was built using as reference the structures of *V. cholera* AphB variant N100E and BenM/BenA from *Acinetobacter* sp. ADP1 (PDB: 3T1B, 4IHT).

Models of wild-type and mutants of RpoB were obtained using I-TASSER, Modeller, and IntFold (McGuffin et al. 2018). I-TASSER and IntFold output were used to further identify potential interacting molecules and their conformation. The predicted structures were inspected with UCSF Chimera and substituted in over 80 of the available structures of the full RNAP complex from *E. coli* at different stages of the transcription process and bound to various σ and transcription-related factors, to elucidate the potential role of mutants. Hybrid models of *P. aeruginosa* RpoB with molecules (DNA, RNA, antibiotic) transferred from *E. coli* structures were further minimized with the AMBER force field using UCSF Chimera. The minimized proposed hybrid model of the *P. aeruginosa* Asp203Glu mutant bound to an open DNA loop and a nascent RNA molecule was subjected to a Molecular Dynamics simulation using NAMD to verify the stability of the proposed model.

## Supplementary Material

Supplementary data are available at *Molecular Biology and Evolution* online.

## Supplementary Material

msac049_Supplementary_Data
