# Supplementary material for: Rapid Decline of Ceftazidime Resistance in Antibiotic-Free and Sublethal Environments Is Contingent on Genetic Background
Source: Mol Biol Evol. 2022 Mar 15;39(3):msac049. doi: 10.1093/molbev/msac049 (PMC8935207; doi:10.1093/molbev/msac049)
Supplement: msac049_Supplementary_Data [file msac049_supplementary_data.docx]

**Supplementary information**

**Supplementary Table 1. Original genetic events in ParR-CAZ, NfxB-CAZ and MDR6-CAZ parental genetic backgrounds.**

| **Parental strain** | **Gene** | **Type** | **Genetic event annotation** |
| --- | --- | --- | --- |
| ParR-CAZ | *parR* | SNP | Glu87Lys |
|  | *ampR* | SNP | Asp135Asn |
|  | Large chromosomal deletion (3409970-3472984) | deletion | Δ63014 bp |
| NfxB-CAZ | *nfxB* | SNP | Phe177Ser |
|  | Arginine, lysine and ornithine decarboxylase (PA14_RS16625) | SNP | Gly23Ala |
|  | Large chromosomal deletion (3220826-3659785) | deletion | Δ438959 bp |
| MDR6-CAZ | *mexC* | SNP | Thr267Ala |
|  | *phoQ* | SNP | Val269Gly |
|  | *frr* | SNP | Ile98Ser |
|  | *pmrB* | SNP | Leu87Gln |
|  | *rpoB* | SNP | Asp203Glu |
|  | *mvfr* | SNP | Arg126Gly |
|  | Large chromosomal deletion (3325314-3631877) | deletion | Δ306563 bp |

**Supplementary Table 2. Tobramycin susceptibility of the genetic backgrounds and population replicates during ALE on antibiotic-free and tobramycin sub-lethal environments.**

| **Treatment** | | **Genetic background** | | **Replicate** | **Tobramycin MIC values (µg/mL)** | | | | | | | | |
| --- | --- | --- | --- | --- | --- | --- | --- | --- | --- | --- | --- | --- | --- |
|  | | |  |  | **0d*** | **7d** | **14d** | **21d** | **28d** | **35d** | **42d** | **49d** | **56d** |
|  | **AB-free** | | **ParR-CAZ** | **1** | 0.5 | 0.5 | 0.5 | 0.75 | 0.75 | 0.5 | 0.38 | 0.5 | 0.5 |
|  |  |  |  | **2** | 0.5 | 0.5 | 0.38 | 0.75 | 0.75 | 0.5 | 0.75 | 0.5 | 0.5 |
|  |  |  |  | **3** | 0.5 | 0.5 | 0.25 | 0.5 | 0.75 | 0.5 | 0.5 | 0.5 | 0.5 |
|  |  |  |  | **4** | 0.5 | 0.5 | 0.5 | 0.5 | 0.5 | 0.5 | 0.5 | 0.5 | 0.5 |
|  |  |  | **NfxB-CAZ** | **1** | 0.5 | 0.25 | 0.5 | 0.5 | 0.75 | 0.5 | 0.38 | 0.38 | 0.5 |
|  |  |  |  | **2** | 0.5 | 0.38 | 0.38 | 1 | 0.5 | 0.5 | 0.5 | 0.38 | 0.38 |
|  |  |  |  | **3** | 0.5 | 0.38 | 0.38 | 0.5 | 0.38 | 0.5 | 0.38 | 0.5 | 0.5 |
|  |  |  |  | **4** | 0.5 | 0.38 | 0.38 | 0.75 | 0.38 | 0.5 | 0.5 | 0.38 | 0.5 |
|  |  |  | **MDR6-CAZ** | **1** | 0.25 | 0.5 | 0.38 | 0.25 | 0.38 | 0.5 | 0.25 | 0.25 | 0.38 |
|  |  |  |  | **2** | 0.25 | 0.38 | 0.5 | 0.5 | 0.5 | 0.5 | 0.25 | 0.25 | 0.38 |
|  |  |  |  | **3** | 0.25 | 0.25 | 0.38 | 0.38 | 0.38 | 0.75 | 0.5 | 0.38 | 0.38 |
|  |  |  |  | **4** | 0.25 | 0.38 | 0.38 | 0.5 | 0.38 | 0.5 | 0.25 | 0.25 | 0.38 |
|  | **TOB**  **1/4** | | **ParR-CAZ** | **1** | 0.5 | 1.5 | 1.5 | 2 | 2 | 2 | 1.5 | 3 | 3 |
|  |  |  |  | **2** | 0.5 | 1.5 | 1.5 | 1.5 | 2 | 2 | 2 | 1.5 | 1.5 |
|  |  |  |  | **3** | 0.5 | 2 | 2 | 2 | 3 | 3 | 2 | 3 | 3 |
|  |  |  |  | **4** | 0.5 | 1.5 | 1.5 | 2 | 3 | 2 | 2 | 2 | 2 |
|  |  |  | **NfxB-CAZ** | **1** | 0.5 | 1 | 1 | 2 | 2 | 2 | 2 | 2 | 3 |
|  |  |  |  | **2** | 0.5 | 1 | 1 | 3 | 2 | 2 | 1.5 | 1 | 1.5 |
|  |  |  |  | **3** | 0.5 | 3 | 3 | 12 | 8 | 8 | 6 | 4 | 4 |
|  |  |  |  | **4** | 0.5 | 1 | 1 | 2 | 1 | 2 | 1.5 | 1.5 | 2 |
|  |  |  | **MDR6-CAZ** | **1** | 0.25 | 0.5 | 0.75 | 0.5 | 0.75 | 1 | 0.5 | 1 | 1 |
|  |  |  |  | **2** | 0.25 | 0.75 | 0.75 | 0.5 | 0.5 | 0.75 | 0.5 | 0.75 | 0.75 |
|  |  |  |  | **3** | 0.25 | 0.5 | 0.5 | 0.5 | 0.5 | 1 | 1 | 1 | 1 |
|  |  |  |  | **4** | 0.25 | 0.75 | 0.75 | 0.75 | 0.75 | 1.5 | 1.5 | 1 | 1.5 |
|  | **TOB**  **1/8** | | **ParR-CAZ** | **1** | 0.5 | 0.5 | 0.5 | 0.75 | 0.75 | 0.75 | 0.5 | 1.5 | 1.5 |
|  |  |  |  | **2** | 0.5 | 0.5 | 0.5 | 1 | 1.5 | 0.75 | 0.75 | 0.75 | 1.5 |
|  |  |  |  | **3** | 0.5 | 0.5 | 0.5 | 1 | 1.5 | 0.75 | 1 | 0.75 | 1.5 |
|  |  |  |  | **4** | 0.5 | 0.5 | 0.5 | 0.75 | 1.5 | 0.5 | 0.75 | 0.75 | 1 |
|  |  |  | **NfxB-CAZ** | **1** | 0.5 | 0.5 | 1 | 1.5 | 2 | 2 | 1.5 | 2 | 3 |
|  |  |  |  | **2** | 0.5 | 0.5 | 0.5 | 0.75 | 1 | 0.75 | 1 | 1 | 1.5 |
|  |  |  |  | **3** | 0.5 | 1 | 1 | 2 | 1.5 | 2 | 1 | 1 | 1.5 |
|  |  |  |  | **4** | 0.5 | 0.5 | 0.5 | 0.75 | 0.5 | 0.75 | 1 | 1.5 | 2 |
|  |  |  | **MDR6-CAZ** | **1** | 0.25 | 0.5 | 0.5 | 0.38 | 0.5 | 0.75 | 0.5 | 0.75 | 0.5 |
|  |  |  |  | **2** | 0.25 | 0.5 | 0.5 | 0.5 | 0.5 | 0.5 | 0.5 | 0.5 | 0.5 |
|  |  |  |  | **3** | 0.25 | 0.5 | 0.75 | 0.5 | 0.5 | 0.75 | 0.5 | 0.75 | 0.75 |
|  |  |  |  | **4** | 0.25 | 0.25 | 0.38 | 0.38 | 0.5 | 0.75 | 0.5 | 0.5 | 0.75 |
|  | **TOB**  **1/16** | | **ParR-CAZ** | **1** | 0.5 | 0.5 | 0.5 | 0.75 | 0.75 | 0.75 | 0.38 | 0.75 | 0.5 |
|  |  |  |  | **2** | 0.5 | 0.5 | 0.38 | 0.75 | 0.75 | 0.5 | 0.75 | 0.5 | 0.75 |
|  |  |  |  | **3** | 0.5 | 0.5 | 0.38 | 0.75 | 0.75 | 0.5 | 0.75 | 0.5 | 0.75 |
|  |  |  |  | **4** | 0.5 | 0.5 | 0.38 | 0.75 | 0.75 | 0.5 | 0.5 | 0.5 | 0.5 |
|  |  |  | **NfxB-CAZ** | **1** | 0.5 | 0.38 | 0.5 | 0.75 | 0.75 | 0.5 | 0.5 | 0.38 | 0.5 |
|  |  |  |  | **2** | 0.5 | 0.38 | 0.38 | 0.75 | 0.5 | 0.5 | 0.5 | 0.5 | 0.5 |
|  |  |  |  | **3** | 0.5 | 0.5 | 0.5 | 0.75 | 0.5 | 0.5 | 0.5 | 0.5 | 0.5 |
|  |  |  |  | **4** | 0.5 | 0.5 | 0.5 | 0.5 | 0.38 | 0.5 | 0.38 | 0.5 | 0.5 |
|  |  |  | **MDR6-CAZ** | **1** | 0.25 | 0.5 | 0.5 | 0.25 | 0.5 | 0.5 | 0.5 | 0.5 | 0.5 |
|  |  |  |  | **2** | 0.25 | 0.38 | 0.5 | 0.5 | 0.5 | 0.75 | 0.5 | 0.5 | 0.5 |
|  |  |  |  | **3** | 0.25 | 0.38 | 0.5 | 0.5 | 0.38 | 0.75 | 0.5 | 0.5 | 0.5 |
|  |  |  |  | **4** | 0.25 | 0.38 | 0.38 | 0.38 | 0.5 | 0.5 | 0.5 | 0.38 | 0.5 |

*MICs at different days of evolution are shown.

**Supplementary Table 3. Ceftazidime susceptibility of the genetic backgrounds and population replicates during ALE on antibiotic-free and tobramycin sub-lethal environments.**

| **Treatment** | **Genetic**  **Background** | **Replicate** | **Ceftazidime MIC values (µg/mL)** | | | | | | | | |
| --- | --- | --- | --- | --- | --- | --- | --- | --- | --- | --- | --- |
|  |  |  | **0d*** | **7d** | **14d** | **21d** | **28d** | **35d** | **42d** | **49d** | **56d** |
| **AB-free** | **ParR-CAZ** | **1** | >256 | >256 | >256 | >256 | >256 | >256 | >256 | >256 | >256 |
|  |  | **2** | >256 | >256 | >256 | >256 | >256 | 1.5/>256** | 2/>256 | 3/>256 | 12 |
|  |  | **3** | >256 | >256 | >256 | >256 | >256 | 1.5/>256 | 1/>256 | 3/>256 | 2/>256 |
|  |  | **4** | >256 | >256 | >256 | >256 | >256 | >256 | >256 | >256 | >256 |
|  | **NfxB-CAZ** | **1** | 8 | 6 | 6 | 6 | 4 | 4 | 4 | 1.5 | 2 |
|  |  | **2** | 8 | 6 | 6 | 8 | 6/2 | 4/1 | 6/1 | 4 | 3 |
|  |  | **3** | 8 | 6 | 6 | 8 | 6 | 3 | 4/2 | 4 | 4 |
|  |  | **4** | 8 | 8 | 6 | 8 | 6 | 4 | 2 | 3 | 3 |
|  | **MDR6-CAZ** | **1** | 12 | 4 | 4 | 6 | 6 | 6 | 6 | 3 | 4 |
|  |  | **2** | 12 | 8 | 8 | 8 | 12 | 12 | 12 | 8 | 8 |
|  |  | **3** | 12 | 8 | 4 | 6 | 6 | 4 | 6 | 4 | 4 |
|  |  | **4** | 12 | 12 | 8 | 8 | 8 | 8 | 12 | 12 | 12 |
| **TOB**  **1/4** | **ParR-CAZ** | **1** | >256 | >256 | >256 | >256 | >256 | >256 | >256 | >256 | >256 |
|  |  | **2** | >256 | >256 | >256 | 4/>256 | 4/>256 | 1.5/>256 | 1.5/8 | 12 | 12 |
|  |  | **3** | >256 | >256 | >256 | 3/>256 | 3/>256 | 4/>256 | 1.5 | 6 | 2 |
|  |  | **4** | >256 | >256 | >256 | 3/>256 | >256 | >256 | >256 | >256 | >256 |
|  | **NfxB-CAZ** | **1** | 8 | 6 | 6 | 8 | 6 | 6 | 4 | 4 | 4 |
|  |  | **2** | 8 | 6 | 6 | 6 | 6 | 6 | 6 | 6 | 4 |
|  |  | **3** | 8 | 2 | 2 | 2 | 1.5 | 0.5/1.5 | 0.5/1.5 | 2 | 2 |
|  |  | **4** | 8 | 6 | 6 | 8 | 8 | 6 | 8 | 6 | 6 |
|  | **MDR6-CAZ** | **1** | 12 | 6 | 4 | 6 | 6 | 8 | 8 | 4 | 6 |
|  |  | **2** | 12 | 12 | 6 | 8 | 12 | 12 | 12 | 8 | 12 |
|  |  | **3** | 12 | 6 | 4 | 6 | 6 | 6 | 6 | 4 | 6 |
|  |  | **4** | 12 | 8 | 8 | 8 | 8 | 8 | 12 | 8 | 6 |
| **TOB**  **1/8** | **ParR-CAZ** | **1** | >256 | >256 | >256 | >256 | >256 | >256 | >256 | >256 | >256 |
|  |  | **2** | >256 | >256 | >256 | >256 | >256 | >256 | >256 | >256 | >256 |
|  |  | **3** | >256 | >256 | >256 | 192/>256 | >256 | 96/>256 | 2/>256 | >256 | >256 |
|  |  | **4** | >256 | >256 | >256 | >256 | >256 | >256 | >256 | >256 | 2/>256 |
|  | **NfxB-CAZ** | **1** | 8 | 8 | 6 | 8 | 6 | 6 | 6 | 6 | 4 |
|  |  | **2** | 8 | 8 | 6 | 12 | 6 | 4/1 | 4/1 | 4 | 4 |
|  |  | **3** | 8 | 6 | 8 | 8 | 8 | 6 | 4 | 4 | 4 |
|  |  | **4** | 8 | 8 | 6 | 6 | 6 | 6 | 6 | 6 | 6 |
|  | **MDR6-CAZ** | **1** | 12 | 8 | 8 | 12 | 12 | 12 | 8 | 8 | 8 |
|  |  | **2** | 12 | 8 | 8 | 8 | 8 | 12 | 12 | 12 | 12 |
|  |  | **3** | 12 | 6 | 6 | 8 | 12 | 8 | 12 | 8 | 12 |
|  |  | **4** | 12 | 8 | 6 | 8 | 8 | 8 | 12 | 8 | 8 |
| **TOB**  **1/16** | **ParR-CAZ** | **1** | >256 | >256 | >256 | >256 | >256 | >256 | 2/>256 | 2/>256 | 3/>256 |
|  |  | **2** | >256 | >256 | >256 | >256 | >256 | >256 | 1.5/>256 | >256 | 2/>256 |
|  |  | **3** | >256 | >256 | >256 | >256 | 2/>256 | >256 | 4/>256 | 4/>256 | 2/>256 |
|  |  | **4** | >256 | >256 | >256 | >256 | 3/>256 | 2/>256 | 2/>256 | 3/>256 | >256 |
|  | **NfxB-CAZ** | **1** | 8 | 8 | 6 | 6 | 4 | 1/4 | 2/6 | 6 | 4 |
|  |  | **2** | 8 | 8 | 6 | 8 | 4 | 1.5/4 | 2/6 | 4 | 4 |
|  |  | **3** | 8 | 6 | 6 | 8 | 6 | 3 | 4 | 4 | 4 |
|  |  | **4** | 8 | 8 | 6 | 6 | 2/6 | 6 | 0.75/6 | 0.75/4 | 4 |
|  | **MDR6-CAZ** | **1** | 12 | 12 | 8 | 12 | 12 | 12 | 8 | 8 | 12 |
|  |  | **2** | 12 | 8 | 4 | 6 | 12 | 8 | 12 | 8 | 8 |
|  |  | **3** | 12 | 6 | 4 | 6 | 6 | 6 | 12 | 6 | 6 |
|  |  | **4** | 12 | 8 | 8 | 8 | 12 | 8 | 12 | 12 | 8 |

*MICs at different days of evolution are shown.

**The MICs for mixed populations are indicated as two different values separated by a forward slash.

**Supplementary Table 4. MIC values (µg/mL) of antibiotics belonging to different structural families in the final populations of *P. aeruginosa* after ALE on antibiotic-free and tobramycin sub-lethal environments.**

| **Treatment** | | **Genetic**  **Background** | | **Replicate** | | **ATM** | | **IPM** | **FOF** | **TET** | **CIP** | **CHL** |
| --- | --- | --- | --- | --- | --- | --- | --- | --- | --- | --- | --- | --- |
|  | **AB-free** | | **ParR-CAZ** | | **Parental** | | >256 | 8 | 24 | 8 | 0.094 | >256 |
|  |  |  |  |  | **1** | | >256 | 8 | 24 | 16 | 0.094 | >256 |
|  |  |  |  |  | **2** | | 16 | 1 | 24 | 16 | 0.094 | >256 |
|  |  |  |  |  | **3** | | 96 | 6 | 32 | 16 | 0.094 | >256 |
|  |  |  |  |  | **4** | | >256 | 4 | 12 | 16 | 0.064 | >256 |
|  |  |  | **NfxB-CAZ** | | **Parental** | | 16 | 6 | 24 | 4 | 0.125 | >256 |
|  |  |  |  |  | **1** | | 8 | 6 | >1024 | 4 | 0.125 | >256 |
|  |  |  |  |  | **2** | | 8 | 3 | 24 | 6 | 0.125 | >256 |
|  |  |  |  |  | **3** | | 6 | 3 | 16 | 4 | 0.094 | >256 |
|  |  |  |  |  | **4** | | 12 | 3 | 24 | 6 | 0.125 | >256 |
|  |  |  | **MDR6-CAZ** | | **Parental** | | 16 | 24 | 12 | 8 | 0.064 | >256 |
|  |  |  |  |  | **1** | | 16 | 1.5 | 24 | 12 | 0.094 | >256 |
|  |  |  |  |  | **2** | | 16 | 8 | 12 | 8 | 0.064 | >256 |
|  |  |  |  |  | **3** | | 8 | 0.5 | 24 | 12 | 0.064 | >256 |
|  |  |  |  |  | **4** | | 16 | 4 | 24 | 6 | 0.064 | >256 |
|  | **TOB**  **1/4** | | **ParR-CAZ** | | **Parental** | | >256 | 8 | 24 | 8 | 0.094 | >256 |
|  |  |  |  |  | **1** | | >256 | 8 | 12 | 4 | 0.064 | >256 |
|  |  |  |  |  | **2** | | 6 | 3 | 12 | 4 | 0.064 | >256 |
|  |  |  |  |  | **3** | | 12 | 0.38 | 32 | 8 | 0.094 | >256 |
|  |  |  |  |  | **4** | | >256 | 8 | 24 | 6 | 0.064 | >256 |
|  |  |  | **NfxB-CAZ** | | **Parental** | | 16 | 6 | 24 | 4 | 0.125 | >256 |
|  |  |  |  |  | **1** | | 12 | 1.5 | 16 | 4 | 0.125 | >256 |
|  |  |  |  |  | **2** | | 12 | 0.5 | 16 | 3 | 0.094 | >256 |
|  |  |  |  |  | **3** | | 6 | 0.38 | >1024 | 16 | 0.125 | >256 |
|  |  |  |  |  | **4** | | 12 | 1.5 | 12 | 3 | 0.094 | >256 |
|  |  |  | **MDR6-CAZ** | | **Parental** | | 16 | 24 | 12 | 8 | 0.064 | >256 |
|  |  |  |  |  | **1** | | 24 | 0.75 | 16 | 6 | 0.064 | >256 |
|  |  |  |  |  | **2** | | 24 | 3 | 16 | 6 | 0.064 | >256 |
|  |  |  |  |  | **3** | | 12 | 0.38 | 12 | 6 | 0.047 | >256 |
|  |  |  |  |  | **4** | | 32 | 4 | 12 | 6 | 0.064 | >256 |
|  | **TOB**  **1/8** | | **ParR-CAZ** | | **Parental** | | >256 | 8 | 24 | 8 | 0.094 | >256 |
|  |  |  |  |  | **1** | | >256 | 6 | 48 | 6 | 0.094 | >256 |
|  |  |  |  |  | **2** | | >256 | 8 | 32 | 8 | 0.094 | >256 |
|  |  |  |  |  | **3** | | 128 | 4 | >1024 | 8 | 0.094 | >256 |
|  |  |  |  |  | **4** | | >256 | 6 | 32 | 8 | 0.094 | >256 |
|  |  |  | **NfxB-CAZ** | | **Parental** | | 16 | 6 | 24 | 4 | 0.125 | >256 |
|  |  |  |  |  | **1** | | 6 | 1.5 | 24 | 4 | 0.125 | >256 |
|  |  |  |  |  | **2** | | 8 | 3 | 32 | 4 | 0.125 | >256 |
|  |  |  |  |  | **3** | | 12 | 2 | 32 | 4 | 0.125 | >256 |
|  |  |  |  |  | **4** | | 8 | 3 | 32 | 4 | 0.125 | >256 |
|  |  |  | **MDR6-CAZ** | | **Parental** | | 16 | 24 | 12 | 8 | 0.064 | >256 |
|  |  |  |  |  | **1** | | 12 | 4 | 16 | 4 | 0.064 | >256 |
|  |  |  |  |  | **2** | | 12 | 4 | 16 | 4 | 0.064 | >256 |
|  |  |  |  |  | **3** | | 12 | 8 | 16 | 3 | 0.064 | >256 |
|  |  |  |  |  | **4** | | 24 | 6 | 12 | 4 | 0.064 | >256 |
|  | **TOB**  **1/16** | | **ParR-CAZ** | | **Parental** | | >256 | 8 | 24 | 8 | 0.094 | 192 |
|  |  |  |  |  | **1** | | >256 | 8 | 16 | 8 | 0.094 | >256 |
|  |  |  |  |  | **2** | | >256 | 8 | 32 | 6 | 0.125 | >256 |
|  |  |  |  |  | **3** | | >256 | 4 | >1024 | 8 | 0.064 | >256 |
|  |  |  |  |  | **4** | | >256 | 6 | 32 | 8 | 0.094 | >256 |
|  |  |  | **NfxB-CAZ** | | **Parental** | | 16 | 6 | 24 | 4 | 0.125 | >256 |
|  |  |  |  |  | **1** | | 12 | 3 | 16 | 3 | 0.125 | >256 |
|  |  |  |  |  | **2** | | 12 | 2 | 24 | 3 | 0.125 | >256 |
|  |  |  |  |  | **3** | | 8 | 2 | 16 | 4 | 0.125 | >256 |
|  |  |  |  |  | **4** | | 8 | 3 | 24 | 3 | 0.125 | >256 |
|  |  |  | **MDR6-CAZ** | | **Parental** | | 16 | 24 | 12 | 8 | 0.064 | >256 |
|  |  |  |  |  | **1** | | 16 | 8 | 12 | 4 | 0.064 | >256 |
|  |  |  |  |  | **2** | | 16 | 6 | 12 | 4 | 0.064 | >256 |
|  |  |  |  |  | **3** | | 24 | 1.5 | 24 | 6 | 0.094 | >256 |
|  |  |  |  |  | **4** | | 8 | 6 | 12 | 3 | 0.064 | 192 |

ATM: aztreonam, IPM: imipenem, FOF: fosfomycin, TET: tetracycline, CIP: ciprofloxacin, CHL: chloramphenicol.

**Supplementary Table 5. Ceftazidime and tobramycin MIC values (µg/mL) of the representative clones isolated from each genetic background and population replicates after 56 days of ALE on antibiotic-free and tobramycin sub-lethal environments.**

| **Treatment** | **Genetic background ParR-CAZ** | | | **Genetic background NfxB-CAZ** | | | **Genetic background MDR6-CAZ** | | |
| --- | --- | --- | --- | --- | --- | --- | --- | --- | --- |
|  | **Clone** | **TOB** | **CAZ** | **Clone** | **TOB** | **CAZ** | **Clone** | **TOB** | **CAZ** |
| **-** | **Parental** | 0.5 | >256 | **Parental** | 0.5 | 8 | **Parental** | 0.25 | 12 |
| **AB-free** | **ParR 1a** | 0.5 | 2 | **NfxB 1a** | 0.25 | 4 | **MDR6 1a** | 0.38 | 4 |
|  | **ParR 1b** | 0.75 | 3 | **NfxB 1b** | 0.19 | 4 | **MDR6 1b** | 0.38 | 3 |
|  | **ParR 1c** | 0.75 | 2 | **NfxB 1c** | 0.19 | 6 | **MDR6 1c** | 0.25 | 4 |
|  | **ParR 1d** | 0.5 | 64 | **NfxB 1d** | 0.19 | 4 | **MDR6 1d** | 0.25 | 6 |
|  | **ParR 2a** | 0.5 | 8 | **NfxB 2a** | 0.38 | 1.5 | **MDR6 2a** | 0.094 | 4 |
|  | **ParR 2b** | 0.38 | 12 | **NfxB 2b** | 0.5 | 6 | **MDR6 2b** | 0.38 | 24 |
|  | **ParR 2c** | 0.75 | 8 | **NfxB 2c** | 0.5 | 1.5 | **MDR6 2c** | 0.094 | 2 |
|  | **ParR 2d** | 0.75 | 8 | **NfxB 2d** | 0.38 | 1.5 | **MDR6 2d** | 0.094 | 2 |
|  | **ParR 3a** | 0.5 | 6 | **NfxB 3a** | 0.75 | 4 | **MDR6 3a** | 0.5 | 6 |
|  | **ParR 3b** | 0.5 | 4 | **NfxB 3b** | 0.25 | 6 | **MDR6 3b** | 0.38 | 6 |
|  | **ParR 3c** | 0.5 | 4 | **NfxB 3c** | 0.5 | 6 | **MDR6 3c** | 0.5 | 6 |
|  | **ParR 3d** | 0.5 | 4 | **NfxB 3d** | 0.75 | 6 | **MDR6 3d** | 0.38 | 6 |
|  | **ParR 4a** | 0.5 | 4 | **NfxB 4a** | 0.75 | 1.5 | **MDR6 4a** | 0.125 | 6 |
|  | **ParR 4b** | 0.75 | >256 | **NfxB 4b** | 0.75 | 6 | **MDR6 4b** | 0.125 | 6 |
|  | **ParR 4c** | 0.5 | >256 | **NfxB 4c** | 0.75 | 4 | **MDR6 4c** | 0.094 | 4 |
|  | **ParR 4d** | 0.5 | >256 | **NfxB 4d** | 0.5 | 6 | **MDR6 4d** | 0.125 | 6 |
| **TOB**  **1/4** | **ParR 1a** | 3 | >256 | **NfxB 1a** | 2 | 6 | **MDR6 1a** | 0.75 | 8 |
|  | **ParR 1b** | 3 | >256 | **NfxB 1b** | 3 | 3 | **MDR6 1b** | 0.5 | 12 |
|  | **ParR 1c** | 3 | >256 | **NfxB 1c** | 2 | 6 | **MDR6 1c** | 1.5 | 8 |
|  | **ParR 1d** | 3 | >256 | **NfxB 1d** | 2 | 1.5 | **MDR6 1d** | 1.5 | 8 |
|  | **ParR 2a** | 2 | 12 | **NfxB 2a** | 2 | 4 | **MDR6 2a** | 0.75 | 6 |
|  | **ParR 2b** | 2 | 12 | **NfxB 2b** | 2 | 2 | **MDR6 2b** | 1 | 6 |
|  | **ParR 2c** | 3 | 8 | **NfxB 2c** | 1.5 | 2 | **MDR6 2c** | 0.75 | 8 |
|  | **ParR 2d** | 3 | 12 | **NfxB 2d** | 2 | 6 | **MDR6 2d** | 0.5 | 6 |
|  | **ParR 3a** | 3 | 3 | **NfxB 3a** | 4 | 2 | **MDR6 3a** | 0.5 | 8 |
|  | **ParR 3b** | 3 | 4 | **NfxB 3b** | 4 | 2 | **MDR6 3b** | 0.75 | 8 |
|  | **ParR 3c** | 3 | 3 | **NfxB 3c** | 4 | 2 | **MDR6 3c** | 0.5 | 8 |
|  | **ParR 3d** | 3 | 4 | **NfxB 3d** | 3 | 2 | **MDR6 3d** | 0.75 | 6 |
|  | **ParR 4a** | 2 | >256 | **NfxB 4a** | 3 | 4 | **MDR6 4a** | 1 | 8 |
|  | **ParR 4b** | 3 | >256 | **NfxB 4b** | 2 | 4 | **MDR6 4b** | 1 | 12 |
|  | **ParR 4c** | 3 | >256 | **NfxB 4c** | 2 | 4 | **MDR6 4c** | 0.5 | 12 |
|  | **ParR 4d** | 2 | >256 | **NfxB 4d** | 2 | 3 | **MDR6 4d** | 1 | 12 |
| **TOB**  **1/8** | **ParR 1a** | 1 | >256 | **NfxB 1a** | 1.5 | 6 | **MDR6 1a** | 0.5 | 12 |
|  | **ParR 1b** | 1 | >256 | **NfxB 1b** | 1.5 | 6 | **MDR6 1b** | 0.38 | 12 |
|  | **ParR 1c** | 1.5 | >256 | **NfxB 1c** | 2 | 6 | **MDR6 1c** | 0.38 | 8 |
|  | **ParR 1d** | 1.5 | >256 | **NfxB 1d** | 1.5 | 6 | **MDR6 1d** | 0.38 | 12 |
|  | **ParR 2a** | 1 | 3 | **NfxB 2a** | 0.75 | 3 | **MDR6 2a** | 0.5 | 12 |
|  | **ParR 2b** | 1.5 | >256 | **NfxB 2b** | 0.75 | 8 | **MDR6 2b** | 0.38 | 12 |
|  | **ParR 2c** | 1.5 | 4 | **NfxB 2c** | 1.5 | 6 | **MDR6 2c** | 0.25 | 12 |
|  | **ParR 2d** | 1.5 | 8 | **NfxB 2d** | 1 | 8 | **MDR6 2d** | 0.38 | 12 |
|  | **ParR 3a** | 1.5 | >256 | **NfxB 3a** | 1.5 | 4 | **MDR6 3a** | 0.38 | 8 |
|  | **ParR 3b** | 1.5 | >256 | **NfxB 3b** | 2 | 6 | **MDR6 3b** | 0.25 | 8 |
|  | **ParR 3c** | 1 | 4 | **NfxB 3c** | 1.5 | 6 | **MDR6 3c** | 0.5 | 12 |
|  | **ParR 3d** | 1.5 | 3 | **NfxB 3d** | 1 | 4 | **MDR6 3d** | 0.38 | 8 |
|  | **ParR 4a** | 1 | >256 | **NfxB 4a** | 1.5 | 6 | **MDR6 4a** | 0.38 | 12 |
|  | **ParR 4b** | 2 | >256 | **NfxB 4b** | 1.5 | 8 | **MDR6 4b** | 0.38 | 12 |
|  | **ParR 4c** | 1.5 | >256 | **NfxB 4c** | 1.5 | 8 | **MDR6 4c** | 0.5 | 8 |
|  | **ParR 4d** | 1.5 | >256 | **NfxB 4d** | 1.5 | 8 | **MDR6 4d** | 0.38 | 12 |
| **TOB**  **1/16** | **ParR 1a** | 0.75 | 4 | **NfxB 1a** | 0.38 | 6 | **MDR6 1a** | 0.5 | 8 |
|  | **ParR 1b** | 0.75 | 6 | **NfxB 1b** | 0.25 | 4 | **MDR6 1b** | 0.25 | 8 |
|  | **ParR 1c** | 0.75 | 6 | **NfxB 1c** | 0.38 | 6 | **MDR6 1c** | 0.38 | 12 |
|  | **ParR 1d** | 0.5 | 4 | **NfxB 1d** | 0.25 | 6 | **MDR6 1d** | 0.38 | 8 |
|  | **ParR 2a** | 0.5 | 3 | **NfxB 2a** | 0.5 | 4 | **MDR6 2a** | 0.38 | 8 |
|  | **ParR 2b** | 0.5 | 3 | **NfxB 2b** | 0.5 | 4 | **MDR6 2b** | 0.38 | 8 |
|  | **ParR 2c** | 0.5 | 2 | **NfxB 2c** | 0.75 | 4 | **MDR6 2c** | 0.25 | 8 |
|  | **ParR 2d** | 0.5 | 2 | **NfxB 2d** | 0.5 | 3 | **MDR6 2d** | 0.38 | 8 |
|  | **ParR 3a** | 0.5 | 6 | **NfxB 3a** | 0.25 | 6 | **MDR6 3a** | 0.38 | 8 |
|  | **ParR 3b** | 0.25 | 24 | **NfxB 3b** | 0.25 | 3 | **MDR6 3b** | 0.25 | 8 |
|  | **ParR 3c** | 0.5 | 3 | **NfxB 3c** | 0.38 | 4 | **MDR6 3c** | 0.25 | 12 |
|  | **ParR 3d** | 0.5 | 3 | **NfxB 3d** | 0.38 | 4 | **MDR6 3d** | 0.38 | 8 |
|  | **ParR 4a** | 0.38 | 6 | **NfxB 4a** | 0.38 | 1 | **MDR6 4a** | 0.25 | 12 |
|  | **ParR 4b** | 0.5 | 6 | **NfxB 4b** | 0.38 | 0.75 | **MDR6 4b** | 0.38 | 12 |
|  | **ParR 4c** | 0.5 | 6 | **NfxB 4c** | 0.38 | 1 | **MDR6 4c** | 0.38 | 8 |
|  | **ParR 4d** | 0.38 | 6 | **NfxB 4d** | 0.5 | 0.75 | **MDR6 4d** | 0.25 | 8 |

**Supplementary Table 6. Relative fitness of the representative clones isolated from each genetic background and population replicates after 56 days of ALE on antibiotic-free and tobramycin sub-lethal environments.**

| **Treatment** | **Clone** | **Relative fitness 56d** |
| --- | --- | --- |
| **AB-free** | **ParR 1a** | 1.02 |
|  | **ParR 2b** | 0.99 |
|  | **ParR 3b** | 1.02 |
|  | **ParR 4a** | 1.16 |
|  | **NfxB 1a** | 1.07 |
|  | **NfxB 2d** | 1.15 |
|  | **NfxB 3b** | 1.03 |
|  | **NfxB 4a** | 1.23 |
|  | **MDR6 1b** | 1.36 |
|  | **MDR6 2c** | 1.19 |
|  | **MDR6 3b** | 1.39 |
|  | **MDR6 4d** | 1.34 |
| **TOB**  **1/4** | **ParR 1a** | 1.15 |
|  | **ParR 2c** | 1.16 |
|  | **ParR 3a** | 1.02 |
|  | **ParR 4b** | 1.12 |
|  | **NfxB 1d** | 1.01 |
|  | **NfxB 2b** | 1.03 |
|  | **NfxB 3c** | 1.34 |
|  | **NfxB 4a** | 1.01 |
|  | **MDR6 1d** | 1.81 |
|  | **MDR6 2b** | 1.40 |
|  | **MDR6 3d** | 1.53 |
|  | **MDR6 4a** | 1.23 |
| **TOB**  **1/8** | **ParR 1c** | 1.27 |
|  | **ParR 2a** | 1.16 |
|  | **ParR 3d** | 1.13 |
|  | **ParR 4b** | 1.22 |
|  | **NfxB 1c** | 1.03 |
|  | **NfxB 2a** | 0.96 |
|  | **NfxB 3a** | 0.89 |
|  | **NfxB 4a** | 1.09 |
|  | **MDR6 1a** | 1.63 |
|  | **MDR6 2a** | 1.61 |
|  | **MDR6 3c** | 1.66 |
|  | **MDR6 4c** | 1.55 |
| **TOB**  **1/16** | **ParR 1a** | 1.04 |
|  | **ParR 2c** | 0.98 |
|  | **ParR 3d** | 0.97 |
|  | **ParR 4a** | 0.96 |
|  | **NfxB 1b** | 1.02 |
|  | **NfxB 2d** | 0.89 |
|  | **NfxB 3b** | 0.86 |
|  | **NfxB 4a** | 0.95 |
|  | **MDR6 1b** | 1.49 |
|  | **MDR6 2a** | 1.52 |
|  | **MDR6 3a** | 1.40 |
|  | **MDR6 4d** | 1.46 |

**Supplementary Table 7. Newly acquired genetic events in antibiotic free medium identified by whole genome sequencing in ParR-CAZ, NfxB-CAZ and MDR6-CAZ parental genetic backgrounds.**

| **Clone** | **Gene annotation** | **Gene** | **Position** | **Type** | **Genetic event** | **Aminoacid change** |
| --- | --- | --- | --- | --- | --- | --- |
| ParR 1a | Large chromosomal deletion |  | 4044383-4205905 | deletion | Δ161522 bp |  |
|  | Aspartate aminotransferase family protein | PA14_RS01595 | 351470 | insertion | 396_398dupTAT | Arg132_Met133insIle |
|  | AmpR | PA14_RS04355 | 934463 | SNP | 491C>T | Pro164Leu |
|  | Lipid-A-disaccharide synthase | PA14_RS06910 | 1476298 | SNP | 470T>G | Val157Gly |
| ParR 2b | Large chromosomal deletion |  | 4053430-4085759 | deletion | Δ32329 bp |  |
|  | *MutL | PA14_RS26710 | 5822293 | SNP | 779A>G | Tyr260Cys |
|  | MarR family transcriptional regulator | PA14_RS08380 | 1795157 | SNP | 401T>C | Leu134Pro |
| ParR 3b | Large chromosomal deletion |  | 4043815-4085759 | deletion | Δ41944 bp |  |
|  | AmpC | PA14_RS04350 | 933771 | deletion | 54delG | Phe19fs |
|  | MarR family transcriptional regulator | PA14_RS08380 | 1794941 | SNP | 185T>G | Val62Gly |
|  | UDP-3-O-acyl-N-acetylglucosamine deacetylase | PA14_RS23345 | 5102405 | SNP | 341T>G | Ile114Ser |
|  | Large chromosomal deletion |  | 4561342-4578431 | deletion | Δ17089 bp |  |
| ParR 4a | Large chromosomal deletion |  | 4061008-4089463 | deletion | Δ28455 bp |  |
|  | *MutL | PA14_RS26710 | 5821181 | SNP | 1891C>T | Arg631Cys |
|  | MarR family transcriptional regulator | PA14_RS08380 | 1794941 | SNP | 185T>G | Val62Gly |
| NfxB 1a | *MutL | PA14_RS26710 | 5821867 | SNP | 1205C>T | Thr402Ile |
|  | AMP-binding protein | PA14_RS12890 | 2735666 | SNP | 326T>A | Leu109Gln |
|  | IscR | PA14_RS05870 | 1253984 | SNP | 149G>T | Arg50Leu |
| NfxB 2d | Large chromosomal deletion |  | 4069637-4112277 | deletion | Δ42640 bp |  |
|  | NusG | PA14_RS03520 | 743288 | SNP | 230A>G | His77Arg |
|  | IscR | PA14_RS05870 | 1254118 |  | 283C>T | Gln95* |
|  | LysR family transcriptional regulator | PA14_RS06570 | 1401547 |  | 259A>C | Ile87Leu |
|  | AMP-binding protein | PA14_RS12890 | 2735563 | insertion | 224dupT | Trp76fs |
|  | LptA | PA14_RS23615 | 5155457 | insertion | 484_489dupCCGCGC | Pro162_Arg163dup |
| NfxB 3b | Large chromosomal deletion |  | 4065354-4092996 | deletion | Δ27642 bp |  |
|  | IscR | PA14_RS05870 | 1254118 | SNP | 283C>T | Gln95* |
|  | Sensor histidine kinase | PA14_RS08745 | 1884933 | SNP | 2269G>A | Glu757Lys |
|  | AMP-binding protein | PA14_RS12890 | 2735464 | SNP | 124C>T | Gln42* |
| NfxB 4a | Large chromosomal deletion |  | 4065354-4092996 | deletion | Δ27642 bp |  |
|  | IscR | PA14_RS05870 | 1254016 | insertion | 182_183dupCG | Gly62fs |
|  | LysR family transcriptional regulator | PA14_RS06570 | 1401177 | SNP | 629A>C | His210Pro |
|  | AMP-binding protein | PA14_RS12890 | 2736196 | SNP | 856G>C | Ala286Pro |
|  | LptA | PA14_RS23615 | 5155457 | insertion | 484_489dupCCGCGC | Pro162_Arg163dup |
| MDR6 1b | RpoB | PA14_RS03545 | 746753 | SNP | 551G>A | Arg184His |
|  | MarR family transcriptional regulator | PA14_RS08380 | 1794956 | SNP | 200T>C | Leu67Pro |
|  | WecC | PA14_RS09470 | 2029335 | SNP | 35G>A | Gly12Asp |
|  | AMP-binding protein | PA14_RS12890 | 2735662 | SNP | 322G>T | Glu108* |
|  | Outer membrane beta-barrel protein | PA14_RS19980 | 4371803 | SNP | 205C>T | Gln69* |
|  | FleS | PA14_RS20380 | 4459714 | SNP | 1046C>T | Thr349Ile |
| MDR6 2c | Large chromosomal deletion |  | 3098123-3168077 | deletion | Δ69954 bp |  |
|  | Large chromosomal deletion |  | 4062112-4193387 | deletion | Δ131275 bp |  |
|  | RpoB | PA14_RS03545 | 746752 | SNP | 550C>T | Arg184Cys |
|  | GtrS | PA14_RS09300 | 1990735 | SNP | 376T>C | Phe126Leu |
|  | AMP-binding protein | PA14_RS12890 | 2736314 | SNP | 974C>T | Ala325Val |
|  | FlhA | PA14_RS18545 | 4062101 | deletion-insertion | 1788_1793delAAGCATinsGGGCGC | SerIle597GlyAla |
|  | GlmM | PA14_RS25685 | 5605712 | SNP | 1122T>G | Asn374Lys |
| MDR6 3b | Large chromosomal deletion |  | 4071108-4093688 | deletion | Δ22580 bp |  |
|  | RpoB | PA14_RS03545 | 746759 | SNP | 557C>T | Ser186Phe |
|  | GtrS | PA14_RS09300 | 1991019 | SNP | 660C>G | Ser220Arg |
|  | Hypothetical protein | PA14_RS09905 | 2129557 | deletion | 559delA | Met187fs |
|  | AMP-binding protein | PA14_RS12890 | 2736579 | SNP | 1239C>G | Tyr413* |
|  | PhoP | PA14_RS19975 | 4371011 | insertion | 312_313insT | Glu106fs |
|  | PIG-L family deacetylase | PA14_RS26995 | 5891411 | SNP | 1013A>C | His338Pro |
|  | Malic enzyme | PA14_RS27215 | 5955876 | SNP | 248C>T | Pro83Leu |
| MDR6 4d | Large chromosomal deletion |  | 4062112-4193387 | deletion | Δ131275 bp |  |
|  | RpoB | PA14_RS03545 | 747623 | SNP | 1421T>A | Val474Glu |
|  | MarR family transcriptional regulator | PA14_RS08380 | 1795000 | SNP | 244C>T | Arg82Cys |
|  | FlhA | PA14_RS18545 | 4062101 | deletion-insertion | 1788_1793delAAGCATinsGGGCGC | SerIle597GlyAla |
|  | PmrA | PA14_RS25820 | 5636510 | insertion | 517_521dupCAACT | Glu175fs |

*When mutators emerged, only those genetic events present in other replicates of the same genetic background have been included to simplify the analysis.

**Supplementary Table 8. Newly acquired genetic events in sub-lethal tobramycin concentration (1/4 of MIC) identified by whole genome sequencing in ParR-CAZ, NfxB-CAZ and MDR6-CAZ parental genetic backgrounds.**

| **Clone** | **Gene annotation** | **Gene** | **Position** | **Type** | **Genetic event** | **Aminoacid change** |
| --- | --- | --- | --- | --- | --- | --- |
| ParR 1a | Aspartate aminotransferase family protein | PA14_RS01595 | 351983 | SNP | 908T>G | Val303Gly |
|  | PtsP | PA14_RS01795 | 393614 | SNP | 845T>A | Val282Glu |
|  | FusA | PA14_RS03565 | 757319 | SNP | 1655A>G | Tyr552Cys |
|  | UvrY | PA14_RS12495 | 2655520 | SNP | 17T>A | Val6Glu |
|  | AMP-binding protein | PA14_RS12890 | 2736410 | SNP | 1070C>T | Pro357Leu |
|  | FleQ | PA14_RS20385 | 4461536 | SNP | 809T>G | Val270Gly |
| ParR 2c | FusA | PA14_RS03565 | 757178 | SNP | 1514T>C | Phe505Ser |
|  | AmpR | PA14_RS04355 | 934087 | SNP | 115C>T | His39Tyr |
|  | WecB | PA14_RS09465 | 2029021 | insertion | 939_940dupGG | Glu314fs |
|  | AMP-binding protein | PA14_RS12890 | 2735802 | deletion-insertion | 462_465delGCGCinsCCTG | GluArg154AspLeu |
|  | FleQ | PA14_RS20385 | 4461270 | SNP | 1075C>T | Pro359Ser |
|  | HPr family phosphocarrier protein | PA14_RS23645 | 5159843 | deletion-insertion | 134_144delGCAAGAGCATC insTCATGGCCGTG | GlyLysSerIle45ValMetAlaVal |
| ParR 3a | FusA | PA14_RS03565 | 757517 | SNP | 1853C>T | Pro618Leu |
|  | PtsP | PA14_RS01795 | 392770 | SNP | c.1A>G | Met1Val |
|  | AmpC | PA14_RS04350 | 933001 | SNP | 824G>A | Trp275* |
|  | Ferredoxin-NADP reductase | PA14_RS08070 | 1736241 | insertion | 313_318dupCTGCCC | Leu105_Pro106dup |
|  | AMP-binding protein | PA14_RS12890 | 2736580 | deletion | 2736515-2736580del |  |
|  | Large chromosomal deletion |  | 4054970-4094607 | deletion | Δ39637 bp |  |
|  | GlmM | PA14_RS25685 | 5606356 | SNP | 478T>C | Phe160Leu |
|  | PycR | PA14_RS29215 | 6392534 | SNP | 137A>G | Asp46Gly |
| ParR 4b | PtsP | PA14_RS01795 | 393614 | SNP | 845T>A | Val282Glu |
|  | FusA | PA14_RS03565 | 757319 | SNP | 1655A>G | Tyr552Cys |
|  | AMP-binding protein | PA14_RS12890 | 2736700 | deletion | 360_361delAT | Ile120fs |
|  | FlhA | PA14_RS18545 | 4063010 | SNP | 884C>T | Ser295Phe |
| NfxB 1d | FusA | PA14_RS03565 | 757670 | SNP | 2006A>G | Tyr669Cys |
|  | AMP-binding protein | PA14_RS12890 | 2736083 | SNP | 743C>T | Pro248Leu |
|  | FliI | PA14_RS20350 | 4453069 | SNP | 842T>C | Ile281Thr |
|  | LptA | PA14_RS23615 | 5155457 | insertion | 484_489dupCCGCGC | Pro162_Arg163dup |
|  | HPr family phosphocarrier protein | PA14_RS23645 | 5159728 | deletion | 21delC | Ile8fs |
| NfxB 2b | FusA | PA14_RS03565 | 757670 | SNP | 2006A>G | Tyr669Cys |
|  | GtrS | PA14_RS09300 | 1991300 | SNP | 941T>A | Leu314Gln |
|  | FlhB | PA14_RS18560 | 4066868 | SNP | 1027C>T | Gln343* |
|  | PhhR | PA14_RS21570 | 4697229 | deletion-insertion | 677_684delCGCTGGAC insGGCGCATG | ProLeuAsp226ArgArgMet |
|  | PhhR | PA14_RS21570 | 4697242 | deletion-insertion | 670_671delATinsGA | Met224Glu |
|  | LptA | PA14_RS23615 | 5155430 | SNP | 448G>A | Gly150Ser |
| NfxB 3c | *MutS | PA14_RS07025 | 1503423 | SNP | 62A>C | His21Pro |
|  | FusA | PA14_RS03565 | 757319 | SNP | 1655A>G | Tyr552Cys |
| NfxB 4a | FusA | PA14_RS03565 | 757670 | SNP | 2006A>G | Tyr669Cys |
|  | FliG | PA14_RS20360 | 4454874 | insertion | 847_848insAC | Met284fs |
|  | HppD | PA14_RS21610 | 4705693 | deletion | 74delC | Asp27fs |
|  | LptA | PA14_RS23615 | 5155482 | SNP | 500T>C | Val167Ala |
|  | PsdR | PA14_RS23810 | 5199859 | SNP | 32G>A | Arg11His |
| MDR6 1d | RpoB | PA14_RS03545 | 746759 | SNP | 557C>T | Ser186Phe |
|  | FusA | PA14_RS03565 | 757312 | SNP | 1648A>G | Lys550Glu |
|  | AMP-binding protein | PA14_RS12890 | 2736756 | insertion | 1417dupC | His473fs |
|  | LasR | PA14_RS18655 | 4085521 | deletion-insertion | 537_538delCCinsTT | Arg180Trp |
|  | OprH | PA14_RS19980 | 4372004 | insertion | 3_4insT | Lys2fs |
|  | FlgB | PA14_RS20485 | 4485309 | deletion | 342delC | Gln115fs |
|  | MexC | PA14_RS24860 | 5427849 | insertion | 20_21insG | Ile7fs |
| MDR6 2b | WecC | PA14_RS09470 | 2030085 | deletion | 792delG | His265fs |
| MDR6 3d | RpoB | PA14_RS03545 | 746759 | SNP | 557C>T | Ser186Phe |
|  | WecC | PA14_RS09470 | 2030085 | insertion | 792dupG | His265fs |
|  | AMP-binding protein | PA14_RS12890 | 2736756 | insertion | 1417dupC | His473fs |
|  | LasR | PA14_RS18655 | 4085482 | SNP | 577A>G | Thr193Ala |
|  | PtsP | PA14_RS01795 | 393248 | SNP | 479C>T | Thr160Ile |
|  | MexI | PA14_RS03860 | 816536 | SNP | 2860G>A | Ala954Thr |
|  | FleS | PA14_RS20380 | 4459697 | SNP | 1063A>G | Thr355Ala |
|  | HPr family phosphocarrier protein | PA14_RS23645 | 5159884 | SNP | 175G>A | Gly59Ser |
|  | PmrB | PA14_RS25825 | 5637915 | deletion | 1236delC | Ala413fs |
| MDR6 4a | FusA | PA14_RS03565 | 757312 | SNP | 1648A>G | Lys550Glu |
|  | Sensor histidine kinase | PA14_RS08745 | 1883969 | SNP | 3233T>C | Leu1078Pro |
|  | AMP-binding protein | PA14_RS12890 | 2736617 | SNP | 1277C>T | Pro426Leu |

*When mutators emerged, only those genetic events present in other replicates of the same genetic background have been included to simplify the analysis.

**Supplementary Table 9. Newly acquired genetic events in sub-lethal tobramycin concentration (1/8 of MIC) identified by whole genome sequencing in ParR-CAZ, NfxB-CAZ and MDR6-CAZ parental genetic backgrounds.**

| **Clone** | **Gene annotation** | **Gene** | **Position** | **Type** | **Genetic event** | **Aminoacid change** |
| --- | --- | --- | --- | --- | --- | --- |
| ParR 1c | PtsP | PA14_RS01795 | 393671 | SNP | 902G>A | Arg301His |
|  | GtrS | PA14_RS09300 | 1991210 | SNP | 851C>T | Thr284Met |
|  | Uvrc | PA14_RS12495 | 2656075 | SNP | 572A>G | Lys191Arg |
|  | AMP-binding protein | PA14_RS12890 | 2736796 | SNP | 1456G>T | Asp486Tyr |
|  | FlhA | PA14_RS18545 | 4063043 | SNP | 851A>T | Gln284Leu |
|  | LasR | PA14_RS18655 | 4085583 | SNP | 476T>C | Leu159Pro |
|  | Acyl-CoA dehydrogenase family protein | PA14_RS22235 | 4843978 | SNP | 517G>A | Glu173Lys |
| ParR 2a | Large chromosomal deletion |  | 4055357-4088445 | deletion | Δ33088 bp |  |
|  | *MutL | PA14_RS26710 | 5821247 | SNP | 1825C>T | Gln609* |
|  | PtsP | PA14_RS01795 | 393965 | SNP | 1196G>A | Gly399Asp |
| ParR 3d | *MutL | PA14_RS26710 | 5822758 | SNP | 314C>A | Ser105* |
|  | AMP-binding protein | PA14_RS12890 | 2736580 | SNP | 1240T>C | Trp414Arg |
|  | LasR | PA14_RS18655 | 4085706 | SNP | 353T>C | Leu118Pro |
|  | PtsP | PA14_RS01795 | 393900 | deletion | 1131Gdel | Met377fs |
|  | Response regulator | PA14_RS21275 | 4636778 | SNP | 974T>C | Ile325Thr |
| ParR 4b | PtsP | PA14_RS01795 | 394603 | SNP | 1834A>C | Thr612Pro |
|  | MarR family transcriptional regulator | PA14_RS08380 | 1794859 | SNP | 103C>T | Gln35* |
|  | AMP-binding protein | PA14_RS12890 | 2736088 | SNP | 748C>T | Pro250Ser |
|  | LasR | PA14_RS18655 | 4085500 | SNP | 559T>C | Trp187Arg |
|  | FlgD | PA14_RS20475 | 4484719-4484758 | deletion | 4484719-4484758del |  |
|  | Response regulator | PA14_RS21275 | 4638244 | deletion | 2441delT | Leu814fs |
|  | HutC | PA14_RS27505 | 6021368 | SNP | 242T>G | Val81Gly |
| NfxB 1c | FusA | PA14_RS03565 | 757675 | SNP | 2011A>G | Thr671Ala |
|  | LptF | PA14_RS05800 | 1239727 | SNP | 533A>C | Gln178Pro |
|  | AMP-binding protein | PA14_RS12890 | 2735534 | SNP | 194T>C | Leu65Pro |
|  | FleQ | PA14_RS20385 | 4461651 | SNP | 694G>A | Gly232Arg |
|  | PhhA | PA14_RS21575 | 4698375 | deletion | 184delC | Gln62fs |
| NfxB 2a | SpuF | PA14_RS01610 | 355962 | SNP | 727A>G | Thr243Ala |
|  | FusA | PA14_RS03565 | 756506 | SNP | 842C>T | Ala281Val |
|  | AMP-binding protein | PA14_RS12890 | 2735534 | SNP | 194T>C | Leu65Pro |
|  | LptA | PA14_RS23615 | 5155457 | insertion | 484_489dupCCGCGC | Pro162_Arg163dup |
|  | Hfq | PA14_RS26700 | 5819866 | SNP | 170A>G | His57Arg |
| NfxB 3a | FusA | PA14_RS03565 | 757312 | SNP | 1648A>G | Lys550Glu |
|  | Sensor histidine kinase | PA14_RS08745 | 1884074 | SNP | 3128G>A | Cys1043Tyr |
|  | Sensor histidine kinase | PA14_RS08745 | 1884931 | SNP | 2271A>C | Glu757Asp |
|  | AMP-binding protein | PA14_RS12890 | 2735534 | SNP | 194T>C | Leu65Pro |
|  | FleR | PA14_RS20375 | 4459239 | SNP | 308A>T | Lys103Met |
|  | FleQ | PA14_RS20385 | 4461299 | SNP | 1046C>T | Ala349Val |
|  | GlmM | PA14_RS25685 | 5606562 | SNP | 272A>G | His91Arg |
| NfxB 4a | Large chromosomal deletion |  | 4070125-4092034 | deletion | Δ21909 bp |  |
|  | FusA | PA14_RS03565 | 757675 | SNP | 2011A>G | Thr671Ala |
|  | PsdR | PA14_RS23810 | 5200227 | SNP | 400C>T | Gln134* |
|  | PilC | PA14_RS23960 | 5236980 | deletion | 999delC | Ser334fs |
|  | GlmM | PA14_RS25685 | 5605824 | SNP | 1010C>T | Thr337Met |
| MDR6 1a | HutC | PA14_RS27505 | 6021368 | SNP | 242T>G | Val81Gly |
| MDR6 2a | HutC | PA14_RS27505 | 6021368 | SNP | 242T>G | Val81Gly |

*When mutators emerged, only those genetic events present in other replicates of the same genetic background have been included to simplify the analysis.

**Supplementary Table 10. Newly acquired genetic events in sub-lethal tobramycin concentration (1/16 of MIC) identified by whole genome sequencing in the ParR-CAZ, NfxB-CAZ and MDR6-CAZ parental genetic backgrounds.**

| **Clone** | **Gene annotation** | **Gene** | **Position** | **Type** | **Genetic event** | **Aminoacid change** |
| --- | --- | --- | --- | --- | --- | --- |
| ParR 1a | Large chromosomal deletion |  | 4056491-4123466 | deletion | Δ66975 bp |  |
|  | AmpR | PA14_RS04355 | 934786 | SNP | 820delG | Glu274fs |
|  | MarR family transcriptional regulator | PA14_RS08380 | 1794941 | SNP | 185T>G | Val62Gly |
|  | AMP-binding protein | PA14_RS12890 | 2736607 | deletion | 1269delC | Ile424fs |
|  | AmpDh3 | PA14_RS21915 | 4772171 | SNP | 418C>T | Pro140Ser |
|  | PilY1 | PA14_RS24625 | 5376608 | SNP | 3455G>A | Trp1152* |
| ParR 2c | Large chromosomal deletion |  | 4050752-4089335 | deletion | Δ38583 bp |  |
|  | GabD | PA14_RS01425 | 314132 | SNP | 470A>G | Asn157Ser |
|  | Class C beta-lactamase PDC-34 | PA14_RS04350 | 933476 | SNP | 349C>T | Gln117* |
|  | PhoH family protein | PA14_RS04985 | 1063555 | insertion | 7_8insC | Gln5fs |
|  | AMP-binding protein | PA14_RS12890 | 2736629 | SNP | 1289T>G | Met430Arg |
|  | ParS | PA14_RS16730 | 3683624 | SNP | 673T>C | Ser225Pro |
|  | GlmM | PA14_RS25685 | 5605592 | SNP | 1242G>T | Glu414Asp |
| ParR 3d | Large chromosomal deletion |  | 4056260-4116111 | deletion | Δ59851 bp |  |
|  | *MutS | PA14_RS07025 | 1502711 | insertion | 758_759insCAGCCTGCG | Arg253_Ser254insSerLeuArg |
|  | AmpR | PA14_RS04355 | 934786 | insertion | 820dupG | Glu274fs |
|  | AMP-binding protein | PA14_RS12890 | 2735490 | SNP | 151A>G | Gln49Arg |
|  | GlmM | PA14_RS25685 | 5605578 | SNP | 1256T>C | Val419Ala |
| ParR 4a | Large chromosomal deletion |  | 4043678-4121446 | deletion | Δ77768 bp |  |
|  | AmpR | PA14_RS04355 | 934301 | SNP | 329T>C | Leu110Pro |
|  | MarR family transcriptional regulator | PA14_RS08380 | 1794941 | SNP | 185T>G | Val62Gly |
|  | AMP-binding protein | PA14_RS12890 | 2735461 | deletion | 122delA | His41fs |
|  | GlmM | PA14_RS25685 | 5606356 | SNP | 478T>C | Phe160Leu |
|  | GlnA | PA14_RS27575 | 6039928 | SNP | 1192T>C | Tyr398His |
| NfxB 1b | Large chromosomal deletion |  | 4065354-4092996 | deletion | Δ27642 bp |  |
|  | IscR | PA14_RS05870 | 1254118 | SNP | 283C>T | Gln95* |
|  | LysR family transcriptional regulator | PA14_RS06570 | 1401585 | SNP | 221A>G | Asn74Ser |
|  | AMP-binding protein | PA14_RS12890 | 2736036 | deletion-insertion | 696_698delGGTinsCGG | MetVal232IleGly |
|  | Malic enzyme | PA14_RS27215 | 5955639 | SNP | 485A>C | Gln162Pro |
| NfxB 2d | Large chromosomal deletion |  | 4065354-4092996 | deletion | Δ27642 bp |  |
|  | Sensor histidine kinase | PA14_RS08745 | 1884620 | SNP | 2582T>A | Leu861Gln |
|  | AcpP | PA14_RS10415 | 2244356 | SNP | 196G>T | Val66Phe |
|  | AMP-binding protein | PA14_RS12890 | 2735797 | deletion | 458delG | Ser153fs |
| NfxB 3a | Large chromosomal deletion |  | 4049059-4194856 | deletion | Δ145797 bp |  |
|  | *MutS | PA14_RS07025 | 1501411 | SNP | 2074G>A | Asp692Asn |
|  | AMP-binding protein | PA14_RS12890 | 2735343 | SNP | 3G>A | Met1Ile |
|  | IscR | PA14_RS05870 | 1253999 | SNP | 164T>C | Val55Ala |
|  | Malic enzyme | PA14_RS27215 | 5955721 | SNP | 403G>A | Glu135Lys |
| NfxB 4d | Large chromosomal deletion |  | 4050958-4106306 | deletion | Δ55348 bp |  |
|  | IscR | PA14_RS05870 | 1253857 | SNP | 22C>T | Arg8Cys |
|  | GtrS | PA14_RS09300 | 1991024 | SNP | 665C>T | Pro222Leu |
|  | AMP-binding protein | PA14_RS12890 | 2735558 | deletion | 222delG | Ile75fs |
| MDR6 1b | Sulfite exporter TauE/SafE family protein | PA14_RS01810 | 397295 | insertion | 219_223dupCGCGG | Val75fs |
|  | RpoB | PA14_RS03545 | 747614 | SNP | 1412T>A | Val471Glu |
|  | AMP-binding protein | PA14_RS12890 | 2735787 | deletion | 448delG | Glu150fs |
|  | FlgH | PA14_RS20455 | 4480572 | SNP | 242C>T | Ala81Val |
|  | PmrA | PA14_RS25820 | 5636469 | SNP | 475C>T | Arg159Cys |
|  | HutC | PA14_RS27505 | 6021450 | SNP | 160C>T | Gln54* |
| MDR6 3a | RpoB | PA14_RS03545 | 746752 | SNP | 550C>T | Arg184Cys |
|  | GtrS | PA14_RS09300 | 1991024 | SNP | 665C>T | Pro222Leu |
|  | AMP-binding protein | PA14_RS12890 | 2736421 | SNP | 1081C>T | Gln361* |
|  | FlgG | PA14_RS20460 | 4480999 | SNP | 646C>T | Gln216* |
|  | GlmM | PA14_RS25685 | 5606562 | SNP | 272A>G | His91Arg |

*When mutators emerged, only those genetic events present in other replicates of the same genetic background have been included to simplify the analysis.

**Supplementary Table 11. Compensatory genetic events newly acquired in ParR-CAZ, NfxB-CAZ and MDR6-CAZ parental genetic backgrounds after 56 days of ALE in antibiotic-free and sub-lethal tobramycin environments.**

| **Gene annotation** | **Gene** | **Ab-free**  **evolved clones** | **Tobramycin 1/4**  **evolved clones** | **Tobramycin 1/8**  **evolved clones** | **Tobramycin 1/16**  **evolved clones** |
| --- | --- | --- | --- | --- | --- |
| AMP-binding protein | PA14_RS12890 | NfxB (1a, 2d, 3b, 4a)  MDR6 (1b, 2c, 3b) | ParR (1a, 2c, 3a, 4b)  NfxB (1d)  MDR6 (1d, 3d, 4a) | ParR (1c, 3d, 4b) | ParR (1a, 2c, 3d, 4a)  NfxB (1b, 2d, 3b, 4a)  MDR6 (1b, 3a) |
| LptA | PA14_RS23615 | NfxB (2d, 4a) | NfxB (1d, 2b ,4a) | NfxB (2a) |  |
| RpoB | PA14_RS03545 | MDR6 (1b, 2c, 3b, 4d) | MDR6 (1d, 3d) |  | MDR6 (1b, 3a) |
| FlhA | PA14_RS18545 | MDR6 (2c, 4d) | ParR (3a, 4b) | ParR (1c, 2a) |  |
| MarR family transcriptional regulator | PA14_RS08380 | ParR (2b, 3b, 4a)  MDR6 (1b, 4d) |  | ParR (4b) | ParR (1a, 4a) |
| AmpR | PA14_RS04355 | ParR (1a) | ParR (2c) |  | ParR (1a, 3d, 4a) |
| AmpC | PA14_R004355 | ParR (3b) |  |  |  |
| IscR | PA14_RS05870 | NfxB (1a, 2d, 3b, 4a) |  |  | NfxB (1b, 3b, 4a) |
| GtrS | PA14_RS09300 | MDR6 (2c, 3b) |  |  | NfxB (4a)  MDR6 (3a) |
| Large chromosonal deletion  (4071108-4085759) |  | ParR (1a, 2b, 3b, 4a)  NfxB (2d, b3, 4a)  MDR6 (1b, c2, 3b) |  |  | ParR (1a, 2c, 3d, 4a)  NfxB (1b, 2d, 3b, 4a) |

**Supplementary Table 12. Newly acquired tobramycin resistance mutations in ParR-CAZ, NfxB-CAZ and MDR6-CAZ parental genetic backgrounds after 56 days of ALE in sub-lethal tobramycin environments.**

| **Gene annotation** | **Gene** | **Tobramycin 1/4**  **evolved clones** | **Tobramycin 1/8**  **evolved clones** | **Tobramycin 1/16**  **evolved clones** |
| --- | --- | --- | --- | --- |
| PtsP | PA14_RS01795 | ParR (1a, 3a, 4b)  MDR6 (3d) | ParR (1c, 2a, 3d, 4b) |  |
| FusA | PA14_RS03565 | ParR (1a, 2c, 3a, 4b)  NfxB (1d, 2b ,4a)  MDR6 (1d, 4a) | NfxB (1c, 2a, 3a, 4a) |  |
| FleQ | PA14_RS20385 | ParR (1a, 2c) | NfxB (1c, 3a) |  |
| HutC | PA14_RS27505 |  | ParR (4b)  MDR6 (1a, 2a) | MDR6 (1b) |
| GlmM | PA14_RS25685 |  | NfxB (3a, 4a) | ParR (2c, 3d, 4a)  MDR6 (3a) |

**Supplementary Table 13. Intragenic compensatory mutations in *ampR* and *rpoB* in ParR-CAZ and MDR6-CAZ parental genetic backgrounds, respectively, after 56 days of ALE in antibiotic-free medium or sublethal tobramycin environments.**

| **Gene** |  | **Treatment** | **Clone** |  | **Position** | **Type** | **Genetic event** | **Aminoacid change** |
| --- | --- | --- | --- | --- | --- | --- | --- | --- |
| ***ampR*** |  | AB-free | ParR 1a |  | 934463 | SNP | 491C>T | Pro164Leu |
|  |  | TOB 1/4 | ParR 2c |  | 934087 | SNP | 115C>T | His39Tyr |
|  |  | TOB 1/16 | ParR 1a |  | 934786 | SNP | 820delG | Glu274fs |
|  |  |  | ParR 3d |  | 934786 | insertion | 820dupG | Glu274fs |
|  |  |  | ParR 4a |  | 934301 | SNP | 329T>C | Leu110Pro |
| ***rpoB*** |  | AB-free | MDR6 1a |  | 746753 | SNP | 551G>A | Arg184His |
|  |  |  | MDR6 2c |  | 746752 | SNP | 550C>T | Arg184Cys |
|  |  |  | MDR6 3b |  | 746759 | SNP | 557C>T | Ser186Phe |
|  |  |  | MDR6 4d |  | 747623 | SNP | 1421T>A | Val474Glu |
|  |  | TOB 1/4 | MDR6 1d |  | 746759 | SNP | 557C>T | Ser186Phe |
|  |  |  | MDR6 3d |  | 746759 | SNP | 557C>T | Ser186Phe |
|  |  | TOB 1/16 | MDR6 1b |  | 747614 | SNP | 1412T>A | Val471Glu |
|  |  |  | MDR6 3a |  | 746752 | SNP | 550C>T | Arg184Cys |
